# Manufacturing, quality control, and GLP-grade preclinical study of nebulized allogenic adipose mesenchymal stromal cells-derived extracellular vesicles

**DOI:** 10.1186/s13287-024-03708-1

**Published:** 2024-04-02

**Authors:** Jing Wang, Zhong-jin Chen, Ze-yi Zhang, Mei-ping Shen, Bo Zhao, Wei Zhang, Ye Zhang, Ji-gang Lei, Cheng-jie Ren, Jing Chang, Cui-li Xu, Meng Li, Yang-yang Pi, Tian-lun Lu, Cheng-xiang Dai, Su-ke Li, Ping Li

**Affiliations:** 1 Cellular Biomedicine Group (Shanghai), Co. Ltd., 85 Faladi Road, Building 3, Zhangjiang, Pudong New Area, 201210 Shanghai China; 2 Daxing Research Institute, University of Science and Technology Beijing, 100083 Beijing, China

**Keywords:** Extracellular vesicles, Adipose mesenchymal stromal cells, ALI/ARDS, Inflammation

## Abstract

**Background:**

Human adipose stromal cells-derived extracellular vesicles (haMSC-EVs) have been shown to alleviate inflammation in acute lung injury (ALI) animal models. However, there are few systemic studies on clinical-grade haMSC-EVs. Our study aimed to investigate the manufacturing, quality control (QC) and preclinical safety of clinical-grade haMSC-EVs.

**Methods:**

haMSC-EVs were isolated from the conditioned medium of human adipose MSCs incubated in 2D containers. Purification was performed by PEG precipitation and differential centrifugation. Characterizations were conducted by nanoparticle tracking analysis, transmission electron microscopy (TEM), Western blotting, nanoflow cytometry analysis, and the TNF-α inhibition ratio of macrophage [after stimulated by lipopolysaccharide (LPS)]. RNA-seq and proteomic analysis with liquid chromatography tandem mass spectrometry (LC–MS/MS) were used to inspect the lot-to-lot consistency of the EV products. Repeated toxicity was evaluated in rats after administration using trace liquid endotracheal nebulizers for 28 days, and respiratory toxicity was evaluated 24 h after the first administration. In vivo therapeutic effects were assessed in an LPS-induced ALI/ acute respiratory distress syndrome (ARDS) rat model.

**Results:**

The quality criteria have been standardized. In a stability study, haMSC-EVs were found to remain stable after 6 months of storage at − 80°C, 3 months at − 20 °C, and 6 h at room temperature. The microRNA profile and proteome of haMSC-EVs demonstrated suitable lot-to-lot consistency, further suggesting the stability of the production processes. Intratracheally administered 1.5 × 10^8^ particles/rat/day for four weeks elicited no significant toxicity in rats. In LPS-induced ALI/ARDS model rats, intratracheally administered haMSC-EVs alleviated lung injury, possibly by reducing the serum level of inflammatory factors.

**Conclusion:**

haMSC-EVs, as an off-shelf drug, have suitable stability and lot-to-lot consistency. Intratracheally administered haMSC-EVs demonstrated excellent safety at the tested dosages in systematic preclinical toxicity studies. Intratracheally administered haMSC-EVs improved the lung function and exerted anti-inflammatory effects on LPS-induced ALI/ARDS model rats.

**Supplementary Information:**

The online version contains supplementary material available at 10.1186/s13287-024-03708-1.

## Introduction

Mesenchymal stromal cells (MSCs) exist in various adult mesenchymal tissues. They are multipotent and have immunomodulatory abilities [1]. Acute respiratory distress syndrome (ARDS) and acute lung injury (ALI) are life threatening clinical syndromes with high morbidity and mortality; however, there are limited effective clinical interventions for the treatment of ALI/ARDS [2]. Preclinical studies in mice, rats, and sheep have shown that MSC treatments inhibit lung damage, reduce inflammation, suppress immune responses, and promote alveolar fluid clearance, suggesting that MSCs can alleviate ARDS/ALI [3–11]. To date, more than 50 trials have been conducted using MSCs as therapeutic agents in ARDS/ALI (ClinicalTrials.gov). Multiple studies have revealed the safety of MSCs in ARDS treatment, and MSCs may reduce the mortality rate of patients with ARDS [12–15]. However, due to their cell size, MSCs are typically intravenously administered, and the effective dose of MSCs is relatively high (5–1.25 × 10^7^/treatment) [16]. It was reported that some of the biological functions of MSCs are mediated by secreted extracellular vesicles (EVs) and mesenchymal stromal cells-derived extracellular vesicles (MSC-EVs) have shown beneficial effects in ARDS treatment [17–19]. EVs are bilayer membrane vesicles secreted by almost all cell types and play important roles in cell–cell communication. EVs are classified by physical characteristics (size or density, such as small EVs, and medium/large EVs); biochemical composition (CD63+ /CD81+ -EVs; etc.); or by their conditions or cells (podocyte EVs, hypoxic EVs, etc.) [20]. EVs enclose a variety of cargos, including lipids, nucleic acids, and proteins, which can be transported among neighboring or distant cells and participate in regulating biological functions [21]. Preclinical studies have shown that MSC-EVs can improve the survival rate, alleviate lung injury, reduce inflammatory cell infiltration and the level of inflammatory cytokines in alveoli, and alleviate pulmonary endothelial barrier injury [22–33]. Approximately 10 clinical trials investigating the efficacy of MSC-EVs in ARDS treatment are underway, and half of these trials are investigating MSC-EV treatment via administered by inhalation. The results of multiple clinical trials have supported the safety and efficacy of haMSC-EVs in treating ARDS [18, 34]. Compared with those administered via the common intravenous route, EVs administered by inhalation can reach high local concentrations more quickly and may lower the effective dose [18]. It has been reported that human umbilical cord MSC-EVs administered via inhalation outperformed those intravenously administered in reducing inflammation in lipopolysaccharide (LPS) induced ALI mice [35].

Although MSCs-derived EVs have shown great therapeutic potential, few EV drugs have entered clinical trials and most of those studies are still in the preclinical stage. There are no marketed EV products yet. Three challenges the industry is facing are (1) large-scale manufacturing, (2) clinical-grade quality control (QC), and (3) undetermined safety. Differential centrifugation, the most commonly used manufacturing method, cannot realize large-scale production; therefore, more scalable methods, such as precipitation or filtration processes, are being developed. Currently, generally acknowledged criteria for EV characterization are summarized in The Minimal Information for Studies of Extracellular Vesicles 2018 (MISEV2018), which was released by the International Society for Extracellular Vesicles (ISEV). Most of the suggestions were limited to academic studies, and clinical-grade QC was not well-researched [20]. EV characterization MISEV2018 focused on includes cell culture methods, morphological identification, quantification by lipids, nucleic acids, and contamination identification. Clinical-grade QC systems and evaluation criteria have been explored in various studies [36–39]. In addition to characterization, the reported QC assays include parental cell characterization, sterile and virus tests, lot-to-lot consistency assays, and potency assays. However, QC systems of different EV products should be constructed based on different parental cell types, EV types and therapeutic approaches, and a feasible and well-defined system is essential for each EV product. Although EVs are generally considered safe, systematic evaluation of EV safety are limited. According to a 14-day acute oral toxicity test of EVs derived from human adipose tissue-derived mesenchymal cells [40], no abnormal differences in animal death, clinical observation, body weight or gross anatomy were observed between the treatment and control groups. Although a small number of clinical trials have also been conducted to test the efficacy and safety of MSC-EVs [19, 41, 42], currently, there are no available preclinical systemic toxicity tests of nebulized-administered haMSC-EVs.

The haMSC-EVs in this study were manufactured from parental seed cells derived from P4 working bank cells (an intermediate product), which have been used for the manufacture of final cell products for phase II clinical trials on the treatment of knee osteoarthritis approved by the China National Medical Association (NMPA) (CXSL1800109). According to the principle of quality by design (QbD), we developed a scalable production process for haMSC-EVs and critical process parameters (CPPs). A list of critical quality control points (CQCPs) and critical quality attributes (CQAs) was also determined to ensure product quality. To determine the shelf life of the products, stability studies under different storage conditions were designed, and the product characteristics at each endpoint were inspected. To verify the lot-to-lot consistency of the product quality, we conducted RNA and protein analyses using multiple EV lots with RNA sequencing and tandem mass tag (TMT)-based liquid chromatography-tandem mass spectrometry (LC‒MS/MS). Four-week repeated toxicity and respiratory toxicity tests were performed in rats to investigate the safety of intratracheally administered haMSC-EVs. The alleviation of inflammation was verified in an ALI/ARDS rat model. In this study, we established an industrial manufacturing and QC system and tested the safety and efficacy of clinical-grade haMSC-EV products. This study supports the possibility of manufacturing large-scale clinical-grade MSC-EVs, with reduced inner-batch variability and excellent safety.

## Materials and methods

### Cell culture

haMSCs were obtained by isolation from healthy donors in accordance with ethical requirements of ethics. The detailed information was previously reported [18]. The haMSCs were passaged to P4 and cryopreserved in liquid nitrogen until use. RAW 264.7 cells were purchased from ATCC and cultured in DMEM (Gibco, Thermo Fisher Scientific) supplemented with 10% FBS at 37 °C in 95% air humidity and 5% CO_2_.

### EV isolation

haMSC-EV isolation was performed as previously described [18]. Briefly, P4 cells from a working cell bank were seeded at density of 1–1.5 × 10^4^/cm^2^ in 2D cell factories and cultured at 37 °C in 95% air humidity and 5% CO_2_. After 2 days of culture, the cells reached 90% confluence, and the complete medium was changed to EV-depleted medium (medium centrifuged at 12,000×*g* for 6 h to deplete EVs). After incubating for 48 h, the conditioned medium was harvested and depleted of cell debris by differential centrifugation. The supernatant was incubated with 12% PEG for 24 h and centrifuged at 3000×*g* for 1 h at 4 °C. The pellet was resuspended in PBS and centrifuged at 120,000×*g* for 70 min to remove free proteins and impurities. The EVs were resuspended in saline. The product was tested for sterility and was confirmed negative for anerobic and aerobic bacteria, mycoplasma contamination (PCR, negative) and endotoxin contamination (< 50 EU/mL). Qualified product aliquots were stored at − 80 °C until use.

### Transmission electron microscopy (TEM)

A total of 20–40 μL of EVs was placed on a carbon-coated copper grid and negatively stained with 2% phosphotungstic acid solution for 10 min. The sample was then dried for 2 min. The grid was observed and photographed under a transmission electron microscope (TecnaiTM G2 Spirit BioTWIN).

### Nanoparticle tracking analysis (NTA)

Measurements of the particle size distribution and concentration were performed with a ZetaView PMX 120 (Particle Metrix) based on NTA. Briefly, the machine was automatically aligned with polystyrene microspheres. EV samples were diluted 1000 times with PBS, and particle movement was analyzed by a ZetaView 8.04.02 SP2.

### NanoFCM

A nanoflow cytometry instrument (NanoFCM Inc.) was used to analyze the lipid-to particle ratio and the expression of transmembrane proteins. The system was calibrated with 250 nm Std FL SiNPs and silica nanospheres 68–155 nm in diameter for concentration and size, respectively. EVs were incubated with AF488-conjugated CD9, AF488-conjugated CD63, AF488-conjugated CD81 antibodies (BioLegend) or PKH67 (Sigma) at 37 °C for 30 min. After being diluted with PBS, the samples were loaded and analyzed following the manufacturer’s instructions.

### Western blot

Cells and EVs were lysed with NP40 lysis buffer (Thermo Fisher Scientific), and the protein concentration was determined via a BCA assay (Beyotime). Immunoblotting was performed following the standard protocol with a Bio-Rad system using the following primary antibodies: CD63 monoclonal antibody (Ts63) (Thermo Fisher Scientific), CD9 monoclonal antibody (Thermo Fisher Scientific), CD81 monoclonal antibody (M38) (Thermo Fisher Scientific), anti-Hsp70 antibody (Abcam), and calnexin rabbit pAb (ABclonal). The secondary antibodies included an anti-mouse IgG HRP-linked antibody (Cell Signaling Technology), and an anti-rabbit IgG HRP-linked antibody (Cell Signaling Technology). The signal was developed by an enhanced chemiluminescent (ECL) kit (Merck).

### Potency assay

EVs from haMSCs have been reported to induce macrophage polarization toward the M2 phenotype and further alleviate inflammation [43]. Based on this knowledge, a potency assay was performed by measuring TNF-α release from RAW264.7 cells after LPS/saline or LPS/haMSC-EVs treatment. Specifically, RAW 264.7 cells were seeded in 96-well plates and cultured in DMEM supplemented with 10% FBS for 24 h. LPS (Beyotime) with saline, EVs or dexamethasone (Solarbio) was added to the wells. The treatment was repeated once after 8 h. The medium was collected after another 16 h and centrifuged at 500×*g* for 5 min to deplete cell debris. The TNF-α concentration was determined by a Mouse TNF-α ELISA Kit (Bio-Techne, R&D Systems), and the inhibition ratio was calculated as (1-concentration of EV group/concentration of saline group) × 100%.

### Stability study design

The effects of storage time and temperature on EV characteristics were studied. Briefly, EV aliquots were stored at − 80 °C and − 20 °C for 1, 2, 3, and 6 months to investigate long-term and accelerated stability. Stress tests were conducted by 1 or 3 freeze‒thaw cycles or by storing the samples at room temperature for 1, 3 or 6 h. EV concentrations, protein marker expression, and the TNF-α inhibition ratio were determined.

### microRNA sequencing and data analysis

Total RNA was isolated from haMSC-EVs with a miRNeasy (Qiagen) and quantified with an RNA 6000 Pico Chip. Total RNA containing the small RNA fraction was converted into cDNA using the TruSeq Small RNA library prep kit (Illumina) following the manufacturer's instructions. The libraries were purified via 6% Novex TBE PAGE (Thermo Fisher Scientific) and quantified via PicoGreen (Thermo Fisher Scientific). The samples were sequenced on a HiSeq 2500 (Illumina) system. Bioinformatic analysis was performed on the Majorbio Cloud (https://cloud.majorbio.com/). Briefly, qualified reads were mapped to the miRbase database for known microRNAs. Unmatched reads were compared against the ncRNA database Rfam to determine noncoding RNA (ncRNA) types. The remaining unmatched reads were considered potential novel microRNAs. Common microRNAs among the 3 lots were further investigated. Their targets were predicted with miRanda, TargetScan and RNAhybrid. The target genes predicted by more than 2 programs were further analyzed.

### Proteomic analysis

haMSC-EVs were lysed in 8 M urea lysis buffer and centrifuged at 12,000×*g* for 30 min at 4 °C to obtain the supernatants. The protein lysates were quantified by BCA Protein Assay Kit (Thermo Fisher Scientific) following the manufacturer's instructions. Reduction and alkylation were performed with TEAB/TCEP buffer and IAM as previously described [44]. The protein was precipitated with acetone and digested with trypsin at 37 °C overnight. After desalting with an Oasis® HLB 96-well plate and an Oasis® MCX elution plate (Waters), the samples were labeled with TMT10plex™ (Thermo Fisher Scientific) and analyzed via liquid chromatography tandem mass spectrometry (LC‒MS/MS) performed on an EASY-nLC 1200 system (Thermo Fisher Scientific) connected to a Q Exactive HF-X quadrupole Orbitrap mass spectrometer (Thermo Fisher Scientific) through a nanoelectrospray ionization source. The raw data files were analyzed using Proteome Discoverer (Thermo Fisher Scientific, Version 2.4) with the UniProt database. The fold change ratio was calculated on the Majorbio Cloud (https://cloud.majorbio.com/) [45], and proteins with a fold change ratio > 1.5 and *P* < 0.05 were defined as differentially expressed proteins.

### GO and KEGG enrichment analysis

Gene ontology (GO) and Kyoto encyclopedia of genes and genomes (KEGG) enrichment analyses were performed with predicted microRNA target genes or proteins against the DAVID database (https://david.ncifcrf.gov/), and the threshold of significance was set at *P* < 0.05.

### Animals

The rats were housed in a specific pathogen-free facility on a 12-h light/dark cycle, at a constant ambient temperature of 20–26 °C and relative humidity of 40–70%. They were fed standard laboratory chow, and water was provided ad libitum. Before dissection, the rats were anesthetized by intraperitoneal injection of ketamine (75–150 mg/kg) and xylazine (5–10 mg/kg) and then exsanguinated and euthanized. Our animal studies adhered to the Animal Research: Reporting of In Vivo Experiments (ARRIVE) guidelines (Additional file 1).

### Toxicity studies of haMSC-EVs by intratracheal administration

The four-week repeated toxicity of haMSC-EVs was studied in SD rats by intratracheal atomization with trace liquid endotracheal nebulizers (HY-LWH03, Beijing YSKD Biotechnology). A total of 120 healthy SD rats (6–9 weeks, Zhejiang Vital River Laboratory Animal Technology Co., Ltd.) were randomly divided into 4 groups according to the grouping module of Provantis (Instem): control, haMSC-EVs-low, haMSC-EVs-medium and haMSC-EVs-high. The treatment groups administered 0, 0.6, 3, or 15 × 10^7^ particles/rat of haMSC-EVs (0.1 mL/rat), respectively. Each group consisted of 15 male and 15 female rats. haMSC-EVs were given once a day for 28 days and the rats were observed for 33 days posttreatment. During the study, the animals were observed once a day for clinical manifestations, behavior and death. The body weights of the rats were measured once a week. At the end of the administration period (D29) and the end of the observation period (D62), the eye tissues, hematology and coagulation indices, serum biochemical markers, immune function, urine, and bronchoalveolar fluid (BALF) of the rats were analyzed. Gross anatomy was also analyzed, and organ coefficients (organ weight/body weight) were determined. Histopathological tests were performed on the organs of the control and haMSC-EVs-high groups.

In addition, changes in the tidal volume (TV), minute volume (MV), and respiratory rate (RR) of the animals within 24 h after the administration of a single haMSC-EVs were monitored in 5 male and 5 female rats. The respiratory parameters (TV, MV, and RR) were recorded and analyzed via a whole-body plethysmography system before and 15 min, 30 min, 45 min, 1 h, 2 h, 4 h, 8 h, and 24 h after administration. All operations were performed under good laboratory practice (GLP) regulations except for the BALF analysis.

### Therapeutic effect of haMSC-EVs in an LPS-induced ALI/ARDS rat model

Eighty healthy male SD rats (5–6 weeks, SPF Biotechnology, Beijing) were randomly divided into 4 groups: control, LPS + Placebo, LPS + haMSC-EVs, and LPS + Dexamethasone. The Control group was administered normal saline, and the other groups were administered 0.2 mL of LPS (6.4 mg/kg) by intratracheal atomizing. At 0 h and 2 h after LPS atomization, the animals in the LPS + haMSC-EVs and LPS + Dexamethasone groups were administered haMSC-EVs (6 × 10^7^ particles/rat/time) or dexamethasone (1.6 mg/kg/time). The control and LPS + Placebo groups were administered an equal volume of normal saline. All of the drugs were administered with trace liquid endotracheal nebulizers. The serum levels of IL-1β, IL-6 and TNF-α were determined at 4 h and 24 h after the first administration. At 24 h, the right lung tissue was lavaged to collect BALF. Then leukocytes, neutrophils, lymphocytes, and monocytes were counted, and the total protein and albumin levels were determined. In addition, the animal body weight and left lung weight were measured, the lung coefficient (left lung wet weight/body weight) was calculated, and the lung histopathology was performed to determine the injury score.

The statistical analysis was performed with SPSS (version 19.0, IBM). The data were analyzed for homogeneity of variance via ANOVA with the LSD test or Dunnett’s post-hoc test. Otherwise, multiple comparisons were analyzed by the Kruskal–Wallis test or Dunnett T3 test. P ≤ 0.05 indicated statistical significance.

## Results

### Clinical-grade haMSC-EVs have suitable lot-to-lot consistency

**Fig. 1:**
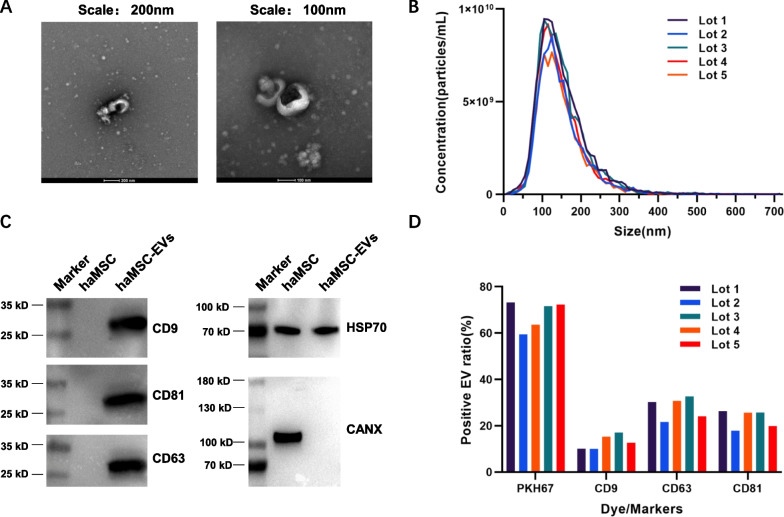
EV characteristics were stable across multiple lots of haMSC-EVs. **A** Representative images of haMSC-EVs at different scales. **B** Size distribution of 5 lots of haMSC-EVs analyzed via NTA. **C** Representative images of the Western blot results for haMSC-EVs and parental cells. The haMSC-EVs were CD9-, CD63-, CD81-, and HSP70-positive and CANX-negative. Full-length blots are presented in Additional file 2: Fig. S1. **D** Marker expression ratios analyzed with nanoflow cytometry

The stability of the production process can usually be reflected by the lot-to-lot consistency of the products. The haMSC-EVs were isolated under the good manufacturing practice (GMP) standard, and CQCPs were determined during drug substance (DS) and drug product (DP) production processes to ensure product safety and quality. The DS production process used conditioned medium of haMSCs to isolate and purify the haMSC-EVs. The DS was stored in saline at high concentration (× 10^10^ particles/mL) after production. The DP production process used saline to dilute haMSC-EVs to 2–8 × 10^8^ particles/3 mL/dose which was ready for use. Specifically, the QC evaluation included (1) safety tests of the cell culture supernatant, including endogenous virus, exogenous virus, and mycoplasma tests; (2) safety tests of the DS (including sterility, mycoplasma, and exogenous virus tests); particle analysis, protein concentration and marker profiling; and (3) safety tests and particle analysis of the DP. The haMSC-EVs used in the DS analysis met all the release criteria. The vesicles in the products showed a cup-shaped morphology in the TEM images, and a representative image is shown in Fig. 1A. The size distributions of 5 lots of drug substances analyzed by NTA are shown in Fig. 1B. The median sizes were between 100 and 150 nm. Based on the Western blot results, compared with the parental cells, the haMSC-EVs were enriched in CD9/63/81 and HSP70 and depleted of CANX (Fig. 1C). To further characterize haMSC-EVs, methodologies to test the marker expression ratios via nanoflow cytometry were developed using CD9, CD63 and CD81 antibodies and a PKH67 molecular probe. The minimum to maximum positive vesicle ratios of 5 batches were 59.4–73.2% for PKH67, 10.0–17.1% for CD9, 21.6–32.6% for CD63 and 17.9–26.3% for CD81. The relative standard deviations (RSDs) between the 5 lots were all less than 30% (9.06% for PHK67, 24.13% for CD9,17.06% for CD63 and 16.85% for CD81). The results suggest that the characteristics of our products are relatively stable. Multiple lots of haMSC-EVs were tested in the potency assay, and the TNF-α inhibition ratios were found to be greater than 30% (31.21% to 56.51% for the batches described above). Based on these existing quality studies, we updated our QC methodologies and summarized the corresponding criteria in Table 1. The quality of the products at the end points of the stability studies was evaluated based on these QC criteria.

**Table 1:** Quality Control of haMSC-EVs

Parameters	Methods	Specifications
Morphology	TEM	Cup-shape or round vesicles at around 100 nm
Size distribution	NTA	Median diameter within 30–160 nm
Concentration		Report as particles/mL
CD9 expression	Western blot	Positive
CD63 expression		Positive
CD81 expression		Positive
HSP70 expression		Positive
CANX expression		Negative
CD9 positive ratio	NanoFCM	≥ 8%
CD63 positive ratio		≥ 20%
CD81 positive ratio		≥ 15%
PKH67 positive ratio		≥ 55%
Potency assay	ELISA	Inhibition ratio of TNF-α release ≥ 30%
Endotoxin	ChP 2020	< 50 EU/mL
Sterile	ChP 2020	Negative
Mycoplasma	qPCR and ChP 2020	Negative
Exogenous virus	ChP 2020	Negative

### haMSC-EVs are stable for DP production and long-term storage

**Fig. 2:**
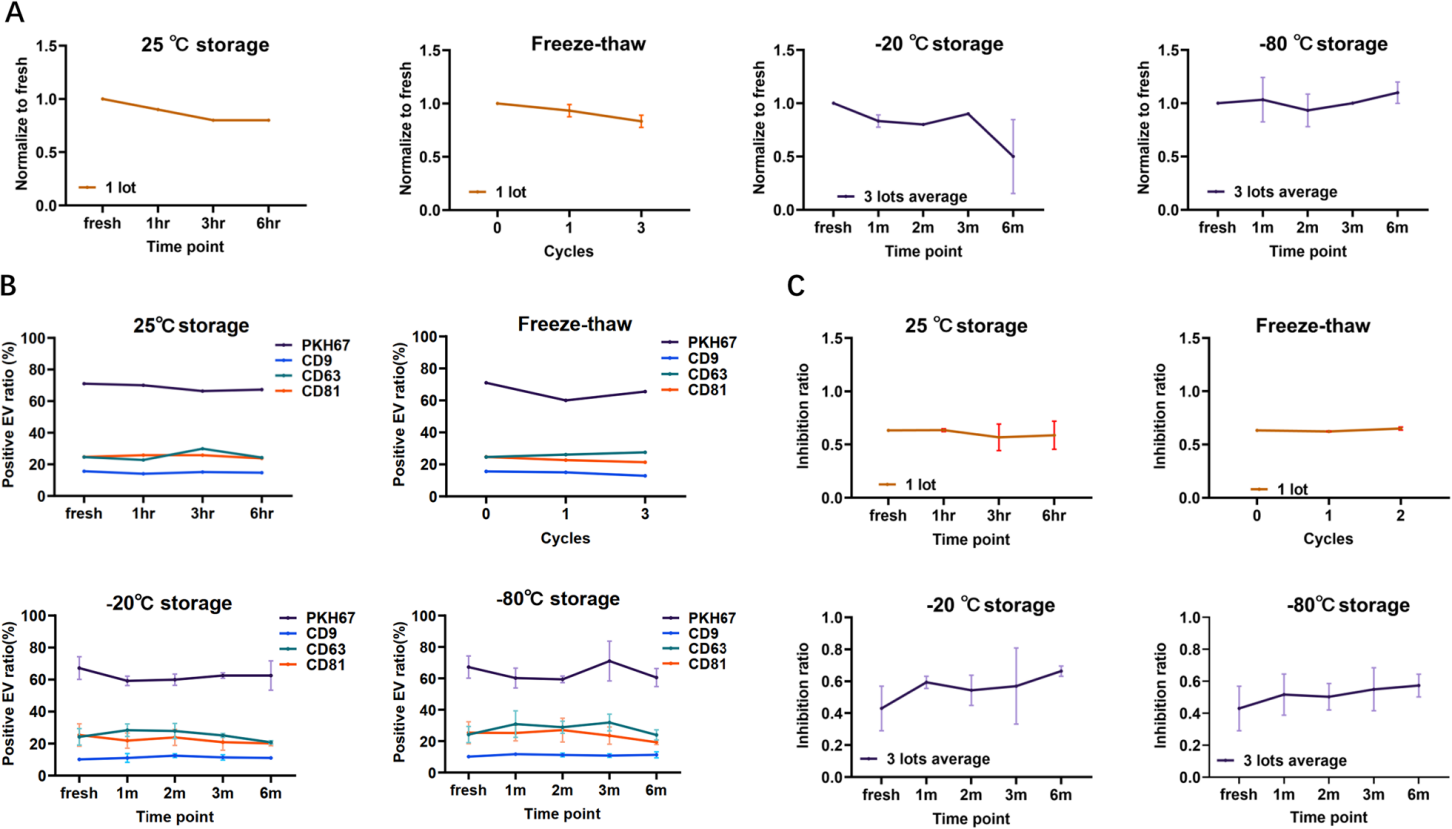
haMSC-EVs were stable during use and long-term storage. **A** NTA of haMSC-EVs stored at 25 °C for 1, 3, or 6 h or freeze‒thawed for 1 or 3 cycles to test the in-use stability and stored at -20 °C or -80 °C for 1, 2, 3, or 6 months to test the storage stability. **B** Membrane marker analysis of haMSC-EVs during use and storage. **C** TNF-α inhibition ratios of haMSC-EVs during stress tests and storage stability tests. 1 lot was taken for each stress test and 3 lots were tested for storage stability tests

Considering that EVs are reportedly unstable at room temperature and sensitive to freeze‒thaw cycles [46], stress tests must be conducted if haMSC-EVs are to be used as drug substances. Thus, we set 3 time points (1 h, 3 h, and 6 h) and 1 or 3 freeze‒thaw cycles to mimic the most extreme scenarios during DP production. The concentration of the particles decreased by less than 20% after 6 h of storage at room temperature or after 3 freeze‒thaw cycles** (**Fig. 2A**)**, while the proportion of membrane marker-positive vesicles decreased by less than 10%** (**Fig. 2B**)**. The TNF-α inhibition ratios decreased by less than 15% after 6 h of storage at room temperature and less than 5% after 3 freeze‒thaw cycles** (**Fig. 2C**)**. The proportions of marker-positive vesicles, and the ratios of TNF-α inhibition of the endpoint samples met the quality criteria **(**Table 1**)**. These results suggested that the normal DP production process does not affect the characteristics or potency of haMSC-EVs. EVs were reported to be most stable when stored at − 80 °C [47, 48]; however, storage at − 20 °C is more common in clinical practice. To investigate the storage stability at − 20 °C, the drug substances were aliquoted and stored in − 20 °C freezer for 1, 2, 3 or 6 months, with samples stored at − 80 °C serving as controls. The NTA data showed that the particle concentrations decreased by no more than 20% after 6 months of storage at − 80 °C and 3 months of storage at − 20 °C. We found that, the particle concentrations decreased by 50% after the samples were stored at − 20 °C for 6 months (Fig. 2A). However, marker-positive vesicle proportions and TNF-α inhibition ratios were not affected and met the quality criteria after 6 months of storage at − 20 °C (Fig. 2B, C), possibly due to delayed degradation of the markers and effectors. Taken together, the results of the stability study suggest that MSC-EVs are stable for drug development and clinical use.

### The microRNA profiles of haMSC-EVs were consistent among different lots

**Fig. 3:**
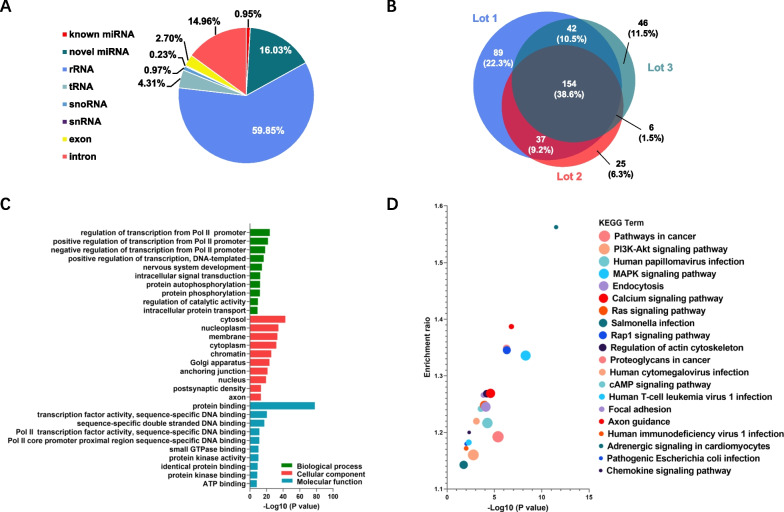
haMSC-EV small RNA sequencing and common microRNA target analysis. **A** Pie chart of small RNA types. **B** Venn diagram of known microRNAs among 3 lots of haMSC-EVs. **C** GO analysis of the predicted targets of 154 common microRNAs. **D** KEGG pathway analysis of the predicted targets of 154 common microRNAs. The dot size represents the number of genes enriched in the corresponding category

Due to their wide regulatory networks, noncoding RNAs, including microRNAs and circular RNAs (circRNAs), have attracted much attention. MicroRNAs have been shown to play important functional roles in MSC-derived EVs [49, 50]. To investigate the microRNA profile of our haMSC-EV product, we constructed small-RNA libraries from 3 lots of haMSC-EVs and performed RNA-seq. We found that haMSC-EVs contain various types of noncoding RNAs, including ribosomal RNA (rRNA), transfer RNA (tRNA), small nucleolar RNA (snoRNA), small nuclear RNA (snRNA) and mRNAs (exons and introns). For the microRNA reads, which accounted for 15.91% of the total reads, 0.95% of the reads were mapped to known microRNAs, and 14.96% were identified as novel microRNAs **(**Fig. 3A**)**. A total of 399 known microRNAs were identified and further compared among the 3 lots of haMSC-EVs, which revealed that 154 (38.6%) microRNAs were shared among the 3 lots of samples, 85 (21.3%) microRNAs were shared between 2 lots, and 160 (40.1%) microRNAs were uniquely identified **(**Fig. 3B**)**. Next, we used miRanda, TargetScan and RNAhybrid to predict the targets of the common 154 microRNAs. A total of 10,098 target genes were predicted by at least 2 software programs and further analyzed.

Gene Ontology (GO) analysis revealed that up to 71 GO biological process (BP) terms were significantly enriched. The BP terms with the greatest significance were regulation of transcription from the RNA polymerase II promoter, nervous system development, and intracellular signal transduction. A total of 105 GO cellular component (CC) terms were significantly enriched, with the cytosol, nucleoplasm and membrane as the 3 CC terms of greatest significance. There were 40 GO molecular function (MF) terms that were significantly enriched, and the 3 most significant MF terms were protein binding, transcription factor activity and sequence-specific double-stranded DNA binding (Fig. 3C).

KEGG pathway analysis revealed that the predicted target genes were associated with 97 KEGG pathways, including pathways involved in cancer, infection, endocytosis, and cell signaling. The 3 most enriched pathways were pathways associated with cancer, the PI3K-Akt signaling pathway, and pathways associated with human papillomavirus infection (Fig. 3D).

The RNA contents in EVs are relatively low and can be easily lost during isolation. According to previous reports, the common microRNA ratio identified in three repeated samples, isolated with commercial kits or TRIzol, is approximately 50% [51]. Our analysis suggested a slightly lower figure of 38.6% for common microRNAs, showing favorable lot-to-lot consistency. Additionally, these common microRNAs were ranked based on TPM (transcripts per million). The content of the top 20 microRNAs was found to be identical among three lots of haMSC-EVs, with a coefficient of variation (CV) of less than 30% (Table 2). These results indicated that the microRNA contents in haMSC-EVs exhibit suitable lot-to-lot consistency under the current production process. The GO and KEGG results revealed that the microRNAs in haMSC-EVs participate in various cell signaling pathways, biological activities, and infection processes.

**Table 2:** List of top 20 known microRNAs detected in 3 lots of haMSC-EVs

Rank	microRNA	Relative content (TPM) × 10^4^			CV (%)
		Lot1	Lot2	Lot3	
1	hsa-let-7b-5p	15.31	13.54	12.89	9
2	hsa-let-7a-5p	14.99	10.97	14.28	16
3	hsa-miR-1290	10.89	11.72	9.90	8
4	hsa-miR-126-5p	7.03	5.43	8.78	24
5	hsa-miR-23a-3p	4.82	4.42	6.43	20
6	hsa-let-7d-3p	4.33	5.38	5.82	15
7	hsa-let-7f-5p	3.47	2.27	3.56	23
8	hsa-miR-92a-3p	2.42	2.56	1.71	20
9	hsa-let-7c-5p	2.30	2.27	1.76	14
10	hsa-miR-574-3p	2.05	1.54	2.13	17
11	hsa-miR-25-3p	1.63	2.23	1.83	16
12	hsa-miR-151a-3p	1.48	1.12	1.52	16
13	hsa-miR-1246	1.65	1.24	1.00	26
14	hsa-miR-21-5p	1.49	0.88	1.27	26
15	hsa-miR-146a-5p	0.97	1.26	0.92	18
16	hsa-miR-24-3p	0.88	1.11	1.03	12
17	hsa-let-7e-5p	1.01	1.18	0.74	22
18	hsa-let-7b-3p	0.99	0.81	0.94	10
19	hsa-miR-1260a	0.62	1.00	0.89	23
20	hsa-miR-1260b	0.55	0.95	0.75	27

*TPM* transcripts per million

### The haMSC-EVs proteome was stable among different lots

**Fig. 4:**
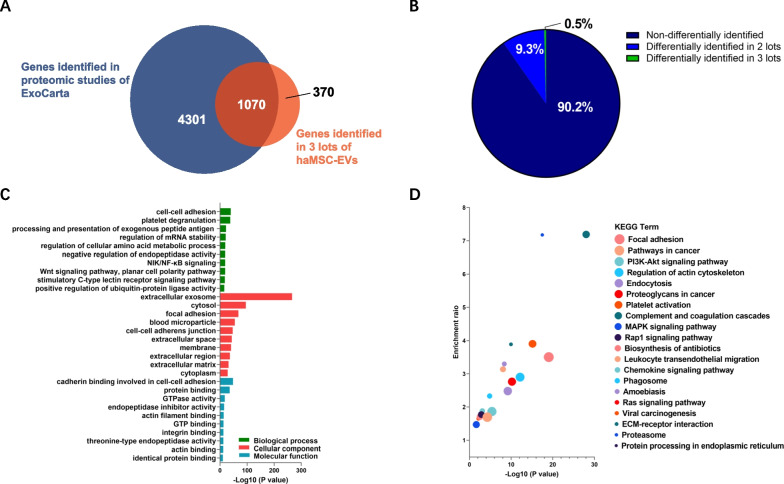
haMSC-EV protein identification, comparison, and functional analysis of the nondifferentially expressed proteins. **A** Venn diagram of genes identified in the ExoCarta database and in 3 lots of haMSC-EVs. **B** Pie chart of differentially and nondifferentially expressed proteins among 3 lots. **C** GO analysis of the nondifferentially expressed proteins. **D** KEGG pathway analysis of the nondifferentially expressed proteins. Dot size represents the gene number enriched in the respective category

The protein cargo that EVs acquire from their parental cells and carry has a large impact on EV function. The proteomic data of EVs vary depending on the parental cells and isolation methods used [52]. Here, we sought to compare the proteomes of our different haMSC-EV lots to determine the consistency of the isolation process and to further understand their mechanism of action (MOA). Total protein was extracted from 3 lots of haMSC-EVs, digested, TMT-labeled, and subjected to LC‒MS/MS analysis. After peptide mapping, 3819 proteins belonging to 1440 genes were identified. The genes were further compared to genes reported in the available EV database ExoCarta, and 1070 genes (74.3%) were found in this database (Fig. 4A). Our haMSC-EV proteomic data were also compared to 100 “exosomal markers” listed in the ExoCarta database, and 81 proteins were found in this list (Table 3). The TMT method enables relative quantification between samples. We compared the relative content of the proteins between every 2 samples. Proteins with more than 1.5-fold differential expression ratio (*P* < 0.05) were defined as differentially expressed proteins, and the others were defined as nondifferentially expressed proteins. The results showed a total of 3446 (90.2%) proteins were nondifferentially expressed, 357 (9.3%) proteins were differentially expressed between 2 lots, and only 16 (0.5%) proteins were differentially expressed among all 3 lots of haMSC-EV samples (Fig. 4B).

**Table 3:** “Exosomal markers” reported in ExoCarta identified in 3 lots of haMSC-EVs

Number	Gene symbol	Gene name	Peptide number identified in haMSC-EVs	Number of times identified in ExoCarta database
1	FLNA	Filamin-A	85	56
2	THBS1	Thrombospondin-1	76	46
3	A2M	Alpha-2-macroglobulin	74	54
4	ALB	Albumin	60	63
5	MYH9	Myosin-9	54	44
6	CLTC	Clathrin heavy chain	41	64
7	HSPA8	Heat shock cognate 71 kDa protein	37	96
8	VCP	Transitional endoplasmic reticulum ATPase	29	62
9	ACTB	Actin, cytoplasmic 1	28	93
10	ACTN4	Alpha-actinin-4	27	51
11	PKM	Pyruvate kinase PKM	27	72
12	PTGFRN	PTGFRN protein	26	46
13	MSN	Moesin	23	62
14	PGK1	Phosphoglycerate kinase 1	23	69
15	HSPA5	Endoplasmic reticulum chaperone BiP	22	58
16	ANXA6	Annexin A6	22	49
17	ALDOA	Fructose-bisphosphate aldolase	20	69
18	ACLY	ATP-citrate synthase	19	48
19	ANXA2	Annexin A2	17	83
20	ATP1A1	Sodium/potassium-transporting ATPase subunit alpha	17	57
21	HSP90AA1	Heat shock protein HSP 90-alpha	17	77
22	LGALS3BP	Galectin-3-binding protein	17	53
23	MVP	Major vault protein	17	44
24	ENO1	Alpha-enolase	16	78
25	EEF2	Elongation factor 2	15	69
26	FASN	Fatty acid synthase	15	66
27	ITGA6	Integrin alpha-6	15	45
28	ITGB1	Integrin beta	15	60
29	UBA1	Ubiquitin-like modifier-activating enzyme 1	15	45
30	RAP1B	Ras-related protein Rap-1b	14	60
31	KPNB1	Importin subunit beta-1	14	48
32	TUBA1C	Tubulin alpha chain	14	47
33	GAPDH	Glyceraldehyde-3-phosphate dehydrogenase	14	95
34	MFGE8	Lactadherin	14	52
35	RAB1A	Ras-related protein Rab-1A	13	45
36	ANXA5	Annexin A5	13	67
37	EEF1A1	Elongation factor 1-alpha	12	71
38	TCP1	T-complex protein 1 subunit alpha	11	44
39	CCT5	CCT-epsilon	11	46
40	HSP90AB1	Heat shock protein HSP 90-beta	11	67
41	RAB8A	Ras-related protein Rab-8A	11	44
42	CCT2	T-complex protein 1 subunit beta	10	56
43	CCT3	T-complex protein 1 subunit gamma (Fragment)	10	46
44	GNAI2	Guanine nucleotide-binding protein G(i) subunit alpha-2	10	53
45	RAB7A	Ras-related protein Rab-7a	10	50
46	STOM	Erythrocyte band 7 integral membrane protein	10	44
47	YWHAZ	14–3-3 protein zeta/delta	10	69
48	CFL1	Cofilin, non-muscle isoform	9	62
49	EZR	Ezrin	9	48
50	LDHB	L-lactate dehydrogenase A chain	9	50
51	YWHAB	14–3-3 protein beta/alpha	9	50
52	LDHA	L-lactate dehydrogenase	9	72
53	SLC3A2	4F2 cell-surface antigen heavy chain	9	57
54	TPI1	Triosephosphate isomerase	9	62
55	PPIA	Peptidyl-prolyl cis–trans isomerase	8	62
56	CD9	Tetraspanin	8	98
57	RAB14	Ras-related protein Rab-14	8	47
58	TFRC	Transferrin receptor protein 1	8	47
59	YWHAQ	14–3-3 protein theta	8	56
60	PFN1	Profilin-1	8	61
61	EHD4	EH domain-containing protein 4	7	51
62	GNB1	guanine nucleotide binding protein (G protein), beta polypeptide 1	7	47
63	GNB2	Guanine nucleotide-binding protein G(I)/G(S)/G(T) subunit beta-2	7	57
64	RAC1	Ras-related C3 botulinum toxin substrate	7	53
65	RAN	GTP-binding nuclear protein Ran	7	46
66	YWHAH	14–3-3 protein eta	7	44
67	PRDX1	Peroxiredoxin-1	7	61
68	CDC42	Cell division control protein 42 homolog	6	55
69	TKT	Transketolase	6	44
70	YWHAG	14–3-3 protein gamma	6	54
71	GNAS	GNAS complex locus isoform 2	5	50
72	RAB5C	Ras-related protein Rab-5C	5	49
73	SDCBP	Syndecan binding protein (Syntenin), isoform CRA_a	5	78
74	AHCY	Adenosylhomocysteinase	4	46
75	RHOA	Transforming protein RhoA	4	52
76	CD63	Tetraspanin	4	82
77	BSG	Basigin	3	45
78	CD81	CD81 antigen	3	64
79	RAB5B	Ras-related protein Rab-5B	3	45
80	LAMP2	Lysosome-associated membrane glycoprotein 2	1	45
81	PRDX2	cDNA FLJ60461, highly similar to Peroxiredoxin-2	1	51

The nondifferentially expressed proteins were subjected to GO and KEGG enrichment analyses. GO analysis indicated that 441 GO BP terms were significantly enriched. The terms with the most significance were cell‒cell adhesion, platelet degranulation and processing, and presentation of exogenous peptide antigen via TAP-dependent MHC class I. A total of 168 GO CC terms were significantly enriched, with the terms of greatest significance being extracellular exosome, cytosol, and focal adhesion. A total of 143 MF GO terms were significantly enriched. The most statistically significant terms were cadherin binding involved in cell‒cell adhesion, protein binding, and GTPase activity (Fig. 4C). KEGG analysis revealed that the proteins were enriched in 67 pathways (*P* < 0.05), and the most significantly enriched pathways were pathways associated with focal adhesion, pathways involved in cancer, and the PI3K-Akt signaling pathway. There were also pathways related to complement and coagulation cascades, bacterial infection, and chemokine signaling pathways, which were highly enriched (Fig. 4D).

Taken together, the EV attributes of our product are supported by the protein profile, as the results agreed well with those of the ExoCarta database. The relative quantification data demonstrated that the protein cargos of our products were consistent among the lots. In addition, gene function analyses revealed the possible mechanism of the well-reported anti-inflammatory effects of haMSC-EVs[6, 43], which are likely mediated by the proteins involved in bacterial infections and complement cascades.

### Four-week repeated toxicity and respiratory toxicity tests in rats to assess the safety of pulmonary haMSC-EV administration

**Fig. 5:**
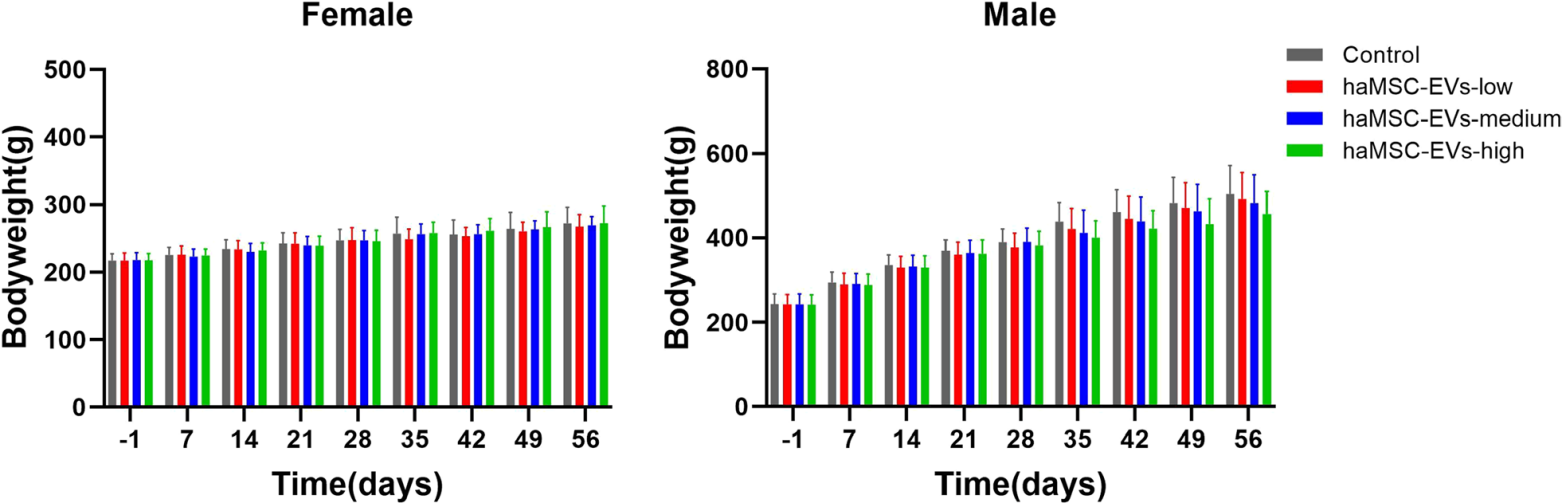
haMSC-EVs at three different doses exhibited no influence on the body weight of SD rats

Quality, safety and efficiency are three essential factors of medicine. There are currently no GLP-grade toxicity data for inhaled EVs. Therefore, we conducted a four-week repeated toxicity study of haMSC-EVs in rats by continuous intratracheal administration to evaluate the safety of haMSC-EVs. A total of 118 animals in the 4 groups (98.3% of the total animals tested) survived to the end of the experiment (D62). One animal in the haMSC-EVs-low group died on D26, and one animal in the haMSC-EVs-high group died on D22. Histopathological examination of the two deceased animals revealed minor lung and kidney lesions, which could be caused by damage during administration or by other disorders. In addition, no significant dose-related abnormalities were found in the clinical symptoms, behaviors, eyes tissues, or urine in any of the animals, including the two deceased animals (data not shown). The average body weight and weight gain of the animals in the treatment groups were not significantly different from those of the Control group. At the end of administration period (D29) and the end of the experiment (D62), no significant administration-related toxicity was observed in any of the organ. The results are shown in Fig. 5, Table 4, and Table 5.

**Table 4:** Effects of haMSC-EVs on female rats’ organ, hematology and coagulation, serum biochemistry and immune function

Time points	D29				D62			
Groups	Control	haMSC-EVs-low	haMSC-EVs-medium	haMSC-EVs-high	Control	haMSC-EVs-low	haMSC-EVs-medium	haMSC-EVs-high
Number of animals	10	9	10	10	5	5	5	5
Organ coefficients (%)								
Brain	0.8296 ± 0.0347	0.7953 ± 0.0725	0.8141 ± 0.0535	0.8264 ± 0.0508	0.7460 ± 0.0248	0.7861 ± 0.0549	0.7774 ± 0.0175	0.7628 ± 0.0700
Heart	0.3834 ± 0.0252	0.3881 ± 0.0328	0.3801 ± 0.0317	0.3766 ± 0.0210	0.3846 ± 0.0212	0.3864 ± 0.0489	0.3497 ± 0.0195	0.3706 ± 0.0217
Liver	2.9306 ± 0.1916	3.0058 ± 0.3383	3.0678 ± 0.2861	2.9715 ± 0.1706	2.9334 ± 0.1904	2.8800 ± 0.1623	2.8182 ± 0.2112	2.6826 ± 0.1439
Spleen	0.2192 ± 0.0092	0.2059 ± 0.0223	0.2152 ± 0.0162	0.2209 ± 0.0247	0.1995 ± 0.0116	0.1945 ± 0.0158	0.1739 ± 0.0162*	0.1771 ± 0.0150
Kidney	0.7123 ± 0.0471	0.6869 ± 0.0508	0.7632 ± 0.0699	0.7507 ± 0.0461	0.7173 ± 0.1032	0.6875 ± 0.0681	0.7036 ± 0.0714	0.7139 ± 0.0287
Thymus	0.1385 ± 0.0285	0.1201 ± 0.0272	0.1529 ± 0.0301	0.1693 ± 0.0249*	0.1138 ± 0.0305	0.1254 ± 0.0373	0.0742 ± 0.0051*	0.1268 ± 0.0214
Uterus	0.2856 ± 0.0755	0.3026 ± 0.1076	0.2543 ± 0.0457	0.2763 ± 0.0829	0.3313 ± 0.1282	0.2914 ± 0.0676	0.2425 ± 0.0527	0.2387 ± 0.0295
Ovary and oviduct	0.0521 ± 0.0070	0.0483 ± 0.0102	0.0531 ± 0.0084	0.0601 ± 0.0118	0.0501 ± 0.0063	0.0548 ± 0.0052	0.0492 ± 0.0098	0.053 ± 0.0037
Adrenal gland	0.0291 ± 0.0040	0.0272 ± 0.0046	0.0269 ± 0.0044	0.028 ± 0.0057	0.0272 ± 0.0059	0.0245 ± 0.0032	0.0261 ± 0.0065	0.0238 ± 0.0039
Thyroid and parathyroid glands	0.0102 ± 0.0025	0.0088 ± 0.0039	0.0099 ± 0.0025	0.0109 ± 0.0036	0.0113 ± 0.0034	0.0112 ± 0.0027	0.0101 ± 0.0020	0.0145 ± 0.0023
Hematology and coagulation indicators								
RBC (× 10^6^/μL)	7.484 ± 0.276	7.691 ± 0.275	7.376 ± 0.306	7.467 ± 0.444	7.840 ± 0.413	7.828 ± 0.081	8.126 ± 0.13	8.122 ± 0.212
Hemoglobin (g/dL)	14.03 ± 0.46	14.42 ± 0.48	13.81 ± 0.54	14.03 ± 0.69	14.58 ± 0.46	14.62 ± 0.19	14.92 ± 0.53	15.28 ± 0.36
Hematocrit	44.14 ± 1.49	45.87 ± 1.58	43.65 ± 1.97	44.13 ± 2.05	44.84 ± 1.75	45.30 ± 0.97	46.64 ± 2.40	46.70 ± 1.05
WBC (× 10^3^/μL)	6.058 ± 1.189	6.023 ± 1.535	6.417 ± 1.512	6.194 ± 1.856	5.168 ± 1.624	5.114 ± 2.530	4.056 ± 1.393	4.330 ± 1.322
NEU%	13.03 ± 6.57	13.28 ± 4.69	15.39 ± 7.28	12.77 ± 2.46	10.38 ± 4.55	9.60 ± 4.56	12.98 ± 2.28	15.42 ± 2.69
LYM%	83.88 ± 7.06	84.42 ± 4.67	82.04 ± 7.80	84.76 ± 2.62	87.42 ± 4.93	87.84 ± 5.52	84.76 ± 2.13	81.22 ± 2.88
MON%	1.12 ± 0.39	0.87 ± 0.29	1.08 ± 0.38	0.86 ± 0.21	0.98 ± 0.33	1.10 ± 0.74	0.76 ± 0.13	1.66 ± 0.83
EOS%	0.32 ± 0.21	0.26 ± 0.29	0.18 ± 0.14	0.22 ± 0.25	0.08 ± 0.08	0.14 ± 0.05	0.18 ± 0.08	0.24 ± 0.09*
BAS%	0.20 ± 0.09	0.20 ± 0.07	0.21 ± 0.03	0.26 ± 0.05	0.26 ± 0.05	0.18 ± 0.08	0.20 ± 0.00	0.20 ± 0.10
LUC%	1.46 ± 0.76	0.96 ± 0.21	1.08 ± 0.32	1.12 ± 0.27	0.86 ± 0.21	1.14 ± 0.39	1.08 ± 0.29	1.30 ± 0.20
Serum biochemical indicators								
ALT (U/L)	36.5 ± 15.4	38.8 ± 27.5	36.6 ± 7.7	39.0 ± 10.3	29.2 ± 5.5	41.8 ± 23.6	28.6 ± 3.8	42.4 ± 25.2
AST (U/L)	118.7 ± 21.0	138.4 ± 61.1	121.1 ± 24.5	135.3 ± 32.1	89.0 ± 13.3	102.6 ± 35.2	93.2 ± 19.8	92.2 ± 22.0
ALP (U/L)	67.3 ± 18.2	64.4 ± 12.6	79.0 ± 29.8	76.4 ± 29.1	55.6 ± 18	51.2 ± 19.4	58.2 ± 22.9	48.8 ± 6.1
CHOL (mmol/L)	1.874 ± 0.360	1.852 ± 0.237	2.011 ± 0.475	1.775 ± 0.411	1.946 ± 0.175	1.928 ± 0.335	2.022 ± 0.474	1.828 ± 0.544
TG (mmol/L)	0.212 ± 0.051	0.190 ± 0.098	0.194 ± 0.065	0.156 ± 0.053	0.152 ± 0.049	0.162 ± 0.089	0.186 ± 0.111	0.152 ± 0.029
GLU (mmol/L)	9.459 ± 1.374	8.982 ± 2.278	9.121 ± 1.425	8.752 ± 1.629	8.500 ± 1.172	7.932 ± 0.919	7.762 ± 2.26	7.430 ± 0.409
BUN (mmol/L)	7.474 ± 0.856	7.298 ± 1.403	8.341 ± 1.817	8.398 ± 1.821	6.488 ± 1.757	6.834 ± 1.701	6.714 ± 0.817	6.482 ± 0.474
CREA (mmol/L)	31.1 ± 2.3	35.0 ± 4.9	31.1 ± 4.7	30.6 ± 3.5	34.2 ± 6.9	31.0 ± 3.2	32.4 ± 3.6	32.8 ± 1.3
TP (g/L)	63.25 ± 3.99	62.72 ± 2.03	63.24 ± 3.48	61.73 ± 4.34	65.78 ± 1.79	67.30 ± 5.55	66.36 ± 1.59	67.46 ± 2.78
ALB (g/L)	47.66 ± 3.94	47.47 ± 3.10	48.05 ± 3.01	46.78 ± 4.16	48.50 ± 2.56	51.68 ± 5.55	50.00 ± 3.43	52.42 ± 2.80
GLO (g/L)	15.59 ± 1.05	15.26 ± 1.86	15.19 ± 1.43	14.95 ± 1.13	17.28 ± 1.97	15.62 ± 0.92	16.36 ± 2.65	15.04 ± 2.00
Immune function								
CD3+ CD4+ (%)	51.519 ± 5.794	45.657 ± 8.165	49.177 ± 5.166	49.549 ± 6.996	63.082 ± 5.461	59.988 ± 5.758	57.948 ± 8.959	67.652 ± 8.211
CD3+ CD8+ (%)	43.037 ± 6.221	48.732 ± 7.394	44.06 ± 4.015	45.13 ± 6.500	34.198 ± 5.178	36.796 ± 5.334	38.92 ± 8.382	30.022 ± 7.914
CD3+ CD4+ /CD3+ CD8+	1.233 ± 0.284	0.976 ± 0.294	1.133 ± 0.217	1.136 ± 0.294	1.901 ± 0.470	1.676 ± 0.398	1.580 ± 0.536	2.496 ± 1.170
CD161+ (%)	0.475 ± 0.384	0.760 ± 0.606	0.345 ± 0.192	0.520 ± 0.193	0.070 ± 0.104	0.110 ± 0.108	0.100 ± 0.061	0.100 ± 0.087
CD45RA+ (%)	56.011 ± 5.617	58.576 ± 6.744	60.710 ± 4.446	54.780 ± 5.155	55.024 ± 5.113	53.080 ± 7.829	53.390 ± 9.754	47.620 ± 7.266
Test of BALF (N = 5)								
WBC (× 10^3^ cells/μL)	1.52 ± 0.96	1.55 ± 1.24	1.33 ± 0.79	0.94 ± 0.64	3.08 ± 1.65	2.00 ± 1.05	2.30 ± 0.74	1.88 ± 0.89
NEU%	17.32 ± 6.44	18.86 ± 7.66	17.10 ± 4.71	14.80 ± 4.03	19.80 ± 5.05	29.12 ± 5.68	27.50 ± 13.57	21.10 ± 5.48
LYM%	69.72 ± 11.07	63.22 ± 18.69	65.84 ± 5.33	74.54 ± 7.22	66.84 ± 7.10	54.04 ± 8.56	56.12 ± 15.11	64.84 ± 9.30
MON%	0.64 ± 0.50	1.34 ± 0.71	2.06 ± 2.56	0.48 ± 0.36	1.40 ± 0.64	2.12 ± 0.91	2.12 ± 0.74	1.76 ± 0.84
EOS%	1.30 ± 0.87	0.96 ± 0.88	1.02 ± 0.63	0.86 ± 0.59	0.72 ± 0.56	1.02 ± 0.62	0.30 ± 0.12	0.74 ± 0.32
BAS%	3.96 ± 2.23	4.02 ± 2.00	2.92 ± 1.52	2.70 ± 1.08	7.28 ± 11.48	4.16 ± 1.54	3.12 ± 0.44	3.54 ± 0.80
LUC%	10.64 ± 5.19	15.10 ± 12.39	13.70 ± 5.79	8.60 ± 4.07	10.52 ± 1.43	13.72 ± 5.17	13.94 ± 4.10	11.62 ± 4.38

Hematology and coagulation indicators: RBC, red blood cell count; WBC, white blood cell count; NEU%, percentage of neutrophils (%); LYM%, percentage of lymphocytes (%); MON%, percentage of monocytes (%); EOS%, percentage of eosinophils (%); BAS%, percentage of basophils (%); LUC%, percentage of large unclassified cells (%). Serum biochemical indicators：ALT, alanine transaminase; AST, aspartate aminotransferase; ALP, alkaline phosphatase; CHOL, total cholesterol; TG, triglyceride; GLU, glucose; BUN, blood urea nitrogen; CREA, creatinine; TP, total protein; ALB, albumin; GLO, Globulin. Data were expressed as mean±SD. * indicates significantly changed after administration (* P ≤ 0.05)

**Table 5:** Effects of haMSC-EVs on male rats’ organ, hematology and coagulation, serum biochemistry and immune function

Time points	D62				D29			
Groups	Control	haMSC-EVs-low	haMSC-EVs-medium	haMSC-EVs-high	Control	haMSC-EVs-low	haMSC-EVs-medium	haMSC-EVs-high
Number of animals	10	10	9	10	5	5	5	5
Organ coefficients (%)								
Brain	0.5845 ± 0.0297	0.5528 ± 0.0271	0.5615 ± 0.0511	0.5665 ± 0.0407	0.4459 ± 0.0295	0.4711 ± 0.0534	0.4672 ± 0.0594	0.486 ± 0.0353
Heart	0.3709 ± 0.0244	0.3604 ± 0.0219	0.3767 ± 0.0172	0.3532 ± 0.0290	0.3244 ± 0.0209	0.331 ± 0.0326	0.3334 ± 0.0295	0.3502 ± 0.0250
Liver	2.8533 ± 0.1564	2.7510 ± 0.1987	3.0338 ± 0.3085	2.8267 ± 0.2462	2.6246 ± 0.1780	2.6831 ± 0.2501	2.7879 ± 0.2925	2.7908 ± 0.2252
Spleen	0.1837 ± 0.0154	0.1783 ± 0.0197	0.1923 ± 0.0212	0.1974 ± 0.0204	0.1516 ± 0.0116	0.1778 ± 0.0291	0.1607 ± 0.0203	0.1951 ± 0.0272*
Kidney	0.7939 ± 0.0639	0.7225 ± 0.0455*	0.8023 ± 0.0657	0.7487 ± 0.0656	0.6467 ± 0.0623	0.6562 ± 0.0505	0.7117 ± 0.0174	0.7038 ± 0.0435
Thymus	0.1535 ± 0.0358	0.1257 ± 0.0291	0.1346 ± 0.0484	0.1212 ± 0.0212	0.0706 ± 0.0161	0.0748 ± 0.0155	0.0905 ± 0.0046	0.0861 ± 0.0291
Testicle	0.8909 ± 0.0575	0.8315 ± 0.0817	0.8606 ± 0.0570	0.8354 ± 0.0610	0.6977 ± 0.1045	0.7019 ± 0.0837	0.661 ± 0.0870	0.7406 ± 0.0390
Epididymis	0.3104 ± 0.0337	0.2945 ± 0.0235	0.2968 ± 0.0282	0.2980 ± 0.0439	0.2808 ± 0.0547	0.2986 ± 0.0197	0.3170 ± 0.0527	0.2998 ± 0.0585
Adrenal gland	0.0165 ± 0.0024	0.0163 ± 0.0028	0.0165 ± 0.0022	0.0157 ± 0.0033	0.0122 ± 0.0029	0.0130 ± 0.0025	0.0109 ± 0.0019	0.0136 ± 0.0027
Thyroid and parathyroid glands	0.0099 ± 0.0026	0.0107 ± 0.0019	0.0122 ± 0.0039	0.0103 ± 0.0033	0.0122 ± 0.0031	0.0120 ± 0.0018	0.0096 ± 0.0026	0.0138 ± 0.0035
Hematology and coagulation indicators								
RBC (× 10^6^/μL)	8.312 ± 0.313	8.164 ± 0.479	8.287 ± 0.317	8.039 ± 0.286	8.792 ± 0.308	8.454 ± 0.488	8.344 ± 0.477	8.454 ± 0.312
Hemoglobin (g/dL)	16.18 ± 0.61	15.55 ± 0.73	15.88 ± 0.53	15.33 ± 0.45**	15.42 ± 0.83	15.34 ± 0.36	15.12 ± 0.65	15.62 ± 0.34
Hematocrit	50.02 ± 1.23	48.97 ± 2.23	49.79 ± 1.85	48.34 ± 1.06	48.26 ± 2.88	47.60 ± 0.69	47.40 ± 2.18	48.92 ± 1.65
WBC (× 10^3^/μL)	9.532 ± 1.705	9.557 ± 1.411	9.331 ± 2.982	10.002 ± 3.081	7.522 ± 1.733	7.598 ± 1.401	6.588 ± 1.616	7.690 ± 1.384
NEU%	10.74 ± 2.80	11.41 ± 4.50	11.12 ± 3.39	12.55 ± 4.71	12.78 ± 5.74	14.62 ± 6.73	15.10 ± 2.69	13.58 ± 1.70
LYM%	85.00 ± 3.67	84.82 ± 5.36	85.68 ± 4.01	83.87 ± 5.03	84.30 ± 5.84	82.10 ± 6.96	82.04 ± 3.94	83.98 ± 1.65
MON%	1.14 ± 0.33	1.02 ± 0.44	1.14 ± 0.45	0.99 ± 0.41	1.12 ± 0.18	1.24 ± 0.35	0.88 ± 0.22	0.96 ± 0.28
EOS%	0.28 ± 0.17	0.30 ± 0.24	0.17 ± 0.07	0.29 ± 0.27	0.58 ± 0.46	0.56 ± 0.36	0.26 ± 0.09	0.18 ± 0.13
BAS%	0.31 ± 0.10	0.33 ± 0.09	0.31 ± 0.09	0.32 ± 0.08	0.26 ± 0.11	0.22 ± 0.04	0.22 ± 0.04	0.22 ± 0.13
LUC%	2.50 ± 1.81	2.15 ± 1.39	1.58 ± 0.78	2.01 ± 1.24	0.96 ± 0.09	1.24 ± 0.28	1.50 ± 1.54	1.14 ± 0.43
Serum biochemical indicators								
ALT (U/L)	33.7 ± 8.5	35.4 ± 6.7	33.4 ± 6.5	34.2 ± 7.9	32.8 ± 4	33.0 ± 3.4	33.0 ± 5.5	32.8 ± 3.3
AST (U/L)	137.0 ± 32.3	154.6 ± 28.4	130.4 ± 26.6	120.0 ± 37.9	115.8 ± 22.1	108.6 ± 18.6	104.8 ± 22.4	95.8 ± 6.2
ALP (U/L)	170.8 ± 29.5	161.5 ± 38.0	156.7 ± 42.1	148.0 ± 31.7	108.6 ± 17.0	113.2 ± 41.4	129.2 ± 14.4	125.0 ± 18.6
CHOL (mmol/L)	1.722 ± 0.355	1.559 ± 0.307	1.682 ± 0.294	1.725 ± 0.250	1.624 ± 0.387	1.868 ± 0.405	1.610 ± 0.163	1.622 ± 0.163
TG (mmol/L)	0.235 ± 0.051	0.260 ± 0.134	0.277 ± 0.126	0.273 ± 0.154	0.212 ± 0.050	0.156 ± 0.064	0.226 ± 0.112	0.170 ± 0.043
GLU (mmol/L)	9.376 ± 1.785	9.064 ± 1.769	9.831 ± 1.693	9.841 ± 1.526	9.156 ± 1.538	8.314 ± 2.288	8.942 ± 1.736	8.262 ± 1.635
BUN (mmol/L)	6.843 ± 1.017	6.332 ± 0.963	7.726 ± 1.488	7.254 ± 1.074	6.716 ± 1.575	6.488 ± 0.623	6.392 ± 0.428	5.762 ± 0.265
CREA (mmol/L)	23.9 ± 2.1	24.8 ± 1.2	24.3 ± 2.7	24.6 ± 4.2	30.2 ± 3.8	27.0 ± 2.7	26.6 ± 2.3	25.6 ± 4.7
TP (g/L)	58.30 ± 2.42	56.27 ± 2.79	57.66 ± 2.76	57.58 ± 0.80	62.50 ± 2.80	60.10 ± 2.10	59.34 ± 1.83	59.04 ± 3.28
ALB (g/L)	42.87 ± 1.60	39.84 ± 2.17**	41.78 ± 1.99	41.28 ± 1.18	42.6 ± 1.83	42.08 ± 1.83	41.62 ± 1.38	41.38 ± 2.06
GLO (g/L)	15.43 ± 1.49	16.43 ± 1.33	15.88 ± 1.50	16.30 ± 1.10	19.90 ± 1.63	18.02 ± 1.77	17.72 ± 1.53	17.66 ± 1.67
Immune function								
CD3+ CD4+ (%)	47.598 ± 8.136	54.128 ± 9.947	52.594 ± 6.782	51.909 ± 8.007	59.270 ± 1.568	61.938 ± 4.647	63.074 ± 8.953	60.318 ± 5.723
CD3+ CD8+ (%)	47.080 ± 6.460	41.566 ± 10.609	41.521 ± 6.489	43.484 ± 7.287	38.466 ± 1.552	35.788 ± 4.737	34.844 ± 8.495	37.270 ± 5.105
CD3+ CD4+ /CD3+ CD8+	1.057 ± 0.385	1.477 ± 0.778	1.326 ± 0.432	1.272 ± 0.527	1.544 ± 0.100	1.770 ± 0.373	1.972 ± 0.84	1.660 ± 0.385
CD161+ (%)	0.520 ± 0.625	0.510 ± 0.631	0.489 ± 0.276	0.891 ± 0.997	0.130 ± 0.091	0.120 ± 0.045	0.200 ± 0.122	0.060 ± 0.042
CD45RA+ (%)	59.875 ± 5.755	58.970 ± 4.948	59.283 ± 6.278	54.596 ± 9.137	54.670 ± 6.799	52.360 ± 5.548	51.290 ± 4.679	54.810 ± 7.242
Test of BALF (N = 5)								
WBC (× 10^3^ cells/μL)	2.63 ± 1.95	1.25 ± 0.93	1.66 ± 0.49	2.82 ± 2.07	4.17 ± 2.42	3.45 ± 2.17	4.26 ± 3.13	3.76 ± 1.87
NEU%	11.54 ± 3.82	19.44 ± 5.40*	9.84 ± 2.01	13.52 ± 3.58	19.60 ± 2.50	20.64 ± 5.58	21.52 ± 7.17	30.22 ± 5.50
LYM%	81.14 ± 5.01	69.84 ± 7.64*	82.54 ± 4.26	79.02 ± 3.73	67.72 ± 4.22	66.82 ± 9.61	64.10 ± 10.69	50.50 ± 5.70**
MON%	0.88 ± 0.23	0.96 ± 0.78	1.68 ± 1.32	0.72 ± 0.24	1.52 ± 0.55	1.9 ± 0.56	1.54 ± 0.61	1.90 ± 0.70
EOS%	0.96 ± 0.5	0.66 ± 0.43	0.60 ± 0.38	0.86 ± 0.74	0.68 ± 0.27	0.82 ± 0.59	0.58 ± 0.26	0.54 ± 0.21
BAS%	2.22 ± 1.30	2.72 ± 0.90	2.04 ± 0.93	2.18 ± 1.18	2.60 ± 1.13	2.84 ± 0.46	2.40 ± 1.01	3.06 ± 1.09
LUC%	4.70 ± 2.76	8.22 ± 3.93	4.04 ± 0.97	4.98 ± 1.69	10.44 ± 2.19	9.86 ± 5.99	12.26 ± 3.97	16.46 ± 5.82

Hematology and coagulation indicators: RBC, red blood cell count; WBC, white blood cell count; NEU%, percentage of neutrophils (%); LYM%, percentage of lymphocytes (%); MON%, percentage of monocytes (%); EOS%, percentage of eosinophils (%); BAS%, percentage of basophils (%); LUC%, percentage of large unclassified cells (%). Serum biochemical indicators：ALT, alanine aminotransferase; AST, aspartate transaminase; ALP, alkaline phosphatase; CHOL, total cholesterol; TG, triglyceride; GLU, glucose; BUN, blood urea nitrogen; CREA, creatinine; TP, total protein; ALB, albumin; GLO, Globulin. Data were expressed as mean±SD. * and ** indicate significantly changed after administration (* P ≤ 0.05, ** P ≤ 0.01)

On the day after the last day of administration (D29), the hemoglobin (HGB) level of male animals in the haMSC-EVs-high group and the albumin (ALB) level of male animals in the haMSC-EVs-low group were slightly lower than those in the Control group (*P* ≤ 0.01 and *P* ≤ 0.01 respectively). Otherwise, there were no obvious abnormalities in hematological indices, coagulation, or serum biochemical indices in the treatment groups (Table 4, Table 5).

The detection of lymphocyte markers, which are immune function indicators in the blood did not significantly differ between the treatment and control groups. Gross anatomy (all groups) and histopathological examination (Control group and haMSC-EVs-high group) revealed no abnormal changes, except for one male animal in the haMSC-EVs-medium group, which exhibited multifocal red discoloration of the lungs by the end of administration (D29). Histopathological examination of this animal showed slight bleeding of the lungs and immune cell infiltration, which could be caused by the operation during intratracheal administration.

We further examined BALF from the tested animals. By the end of the administration period (D29), the percentage of neutrophils was significantly higher (*P* ≤ 0.05), and the percentage of lymphocytes was significantly lower (*P* ≤ 0.05) in the haMSC-EVs-low group than in the Control group. By the end of the experiment (D62), the percentage of lymphocytes in the haMSC-EVs-high group was significantly lower than that in the control group (*P* ≤ 0.01). However, the absolute counts of neutrophils and lymphocytes did not significantly change in any animals of the treatment groups compared to those in the Control group (Additional file 3: Table S1, S2). Therefore, we presume that the changes in neutrophil and lymphocyte percentages could be due to normal physiological variation. The remaining indicators did not change significantly. The results are shown in Table 4 and 5.

Animal respiratory function indicators were also examined. The TV in each group was occasionally increased at different time points (*P* ≤ 0.05 or *P* ≤ 0.01) after administration, but the difference was not time- or dose- dependent. Other indicators were within the normal range after administration. Moreover, there were no significant differences among the groups at any of the detection time points. The results are shown in Table 6.

**Table 6:** Effects of haMSC-EVs on respiratory function in rats

Genders	Groups	Indicators		Before administration		After administration													
						15 min		30 min		45 min		1 h		2 h		4 h		8 h	24 h
Female	Control	TV (mL)		0.92 ± 0.06		1.18 ± 0.10**		1.11 ± 0.10**		0.99 ± 0.03		0.98 ± 0.04		1.05 ± 0.12		1.01 ± 0.07		1.10 ± 0.10**	1.07 ± 0.08*
		MV (mL)		161.4 ± 96.28		159.8 ± 26.58		109.4 ± 15.06		133.6 ± 53.28		121.4 ± 18.89		107.4 ± 11.91		119.0 ± 35.27		126.0 ± 25.15	147.0 ± 70.04
		RR (bpm)		192.0 ± 126.68		144.6 ± 25.81		101.8 ± 20.41		147.2 ± 67.28		133.8 ± 25.31		108.2 ± 14.27		126.4 ± 51.64		122.4 ± 31.00	140.8 ± 67.45
	haMSC-EVs-low	TV (mL)		0.97 ± 0.05		1.16 ± 0.11**		1.04 ± 0.07		0.92 ± 0.09		0.98 ± 0.04		1.00 ± 0.08		1.05 ± 0.04		1.07 ± 0.07	1.06 ± 0.12
		MV (mL)		119.8 ± 31.45		151.6 ± 32.46		113.8 ± 20.4		112.4 ± 46.69		109.0 ± 18.28		124.8 ± 33.91		124.6 ± 32.05		139.6 ± 50.09	167.2 ± 55.37
		RR (bpm)		133.00 ± 45.01		146.00 ± 41.03		113.20 ± 25.33		136.60 ± 89.70		115.60 ± 29.36		134.60 ± 51.00		126.00 ± 37.28		142.40 ± 52.30	175.00 ± 80.83
	haMSC-EVs-medium	TV (mL)		0.99 ± 0.04		15.41 ± 31.98		1.11 ± 0.17		1.00 ± 0.13		0.99 ± 0.06		1.00 ± 0.14		1.09 ± 0.07		1.06 ± 0.07	1.13 ± 0.11
		MV (mL)		165.60 ± 64.79		506.40 ± 837.46		126.00 ± 29.35		127.00 ± 38.44		134.80 ± 36.09		113.80 ± 35.65		143.40 ± 51.56		117.60 ± 27.90	149.40 ± 23.80
		RR (bpm)		183.80 ± 74.88		128.80 ± 24.99		123.00 ± 41.58		134.40 ± 59.86		144.20 ± 41.58		117.20 ± 27.33		141.00 ± 54.41		117.40 ± 27.18	147.60 ± 37.55
	haMSC-EVs-high	TV (mL)		0.96 ± 0.08		1.22 ± 0.11**		1.10 ± 0.11		0.96 ± 0.07		0.99 ± 0.09		1.03 ± 0.11		1.04 ± 0.11		1.06 ± 0.11	1.15 ± 0.10*
		MV (mL)		135.80 ± 60.32		141.40 ± 11.99		120.60 ± 31.37		139.40 ± 67.88		127.00 ± 32.72		108.20 ± 15.25		107.20 ± 14.69		129.20 ± 40.53	136.20 ± 33.41
		RR (bpm)		155.00 ± 78.58		124.00 ± 23.57		113.80 ± 33.08		162.00 ± 89.15		136.40 ± 31.69		111.80 ± 30.96		110.20 ± 21.75		130.80 ± 42.61	127.60 ± 35.86
Male	Control	TV (mL)		0.96 ± 0.15		1.11 ± 0.17		1.08 ± 0.21		1.03 ± 0.18		1.02 ± 0.18		0.99 ± 0.15		1.07 ± 0.13		1.05 ± 0.16	1.06 ± 0.13
		MV (mL)		129.00 ± 35.92		138.60 ± 17.62		133.00 ± 30.14		115.80 ± 23.61		123.00 ± 26.18		106.80 ± 11.10		131.80 ± 30.41		129.40 ± 39.76	123.00 ± 7.28
		RR (bpm)		144.20 ± 31.07		132.00 ± 18.88		132.20 ± 20.12		115.60 ± 18.72		129.00 ± 41.18		113.20 ± 14.29		131.40 ± 25.31		128.20 ± 31.00	119.80 ± 13.20
	haMSC-EVs-low	TV (mL)		0.86 ± 0.05		1.17 ± 0.11**		1.02 ± 0.08**		0.92 ± 0.11		0.93 ± 0.06		0.91 ± 0.10		0.91 ± 0.10		0.97 ± 0.08	0.92 ± 0.11
		MV (mL)		144.40 ± 83.16		138.60 ± 15.36		121.60 ± 22.21		105.80 ± 6.83		147.80 ± 30.29		119.00 ± 32.28		132.60 ± 55.08		144.60 ± 34.82	113.20 ± 13.52
		RR (bpm)		176.00 ± 105.08		124.60 ± 11.44		122.60 ± 28.45		115.60 ± 7.77		166.80 ± 32.27		131.20 ± 20.18		155.60 ± 60.31		166.60 ± 50.59	126.60 ± 7.02
	haMSC-EVs-medium	TV (mL)		0.97 ± 0.09		1.18 ± 0.14		1.12 ± 0.14		1.06 ± 0.10		1.05 ± 0.14		0.94 ± 0.07		1.04 ± 0.08		1.04 ± 0.17	1.06 ± 0.12
		MV (mL)		150.00 ± 51.04		145.20 ± 20.71		136.40 ± 49.54		110.20 ± 9.09		115.00 ± 6.60		119.60 ± 20.56		116.80 ± 16.30		139.40 ± 27.33	131.40 ± 29.81
		RR (bpm)		168.80 ± 69.03		125.40 ± 19.24		118.60 ± 29.60		105.20 ± 11.12		111.20 ± 13.31		133.20 ± 32.92		117.80 ± 21.92		147.80 ± 49.94	124.40 ± 16.10
	haMSC-EVs-high	TV (mL)		0.87 ± 0.06		1.10 ± 0.12*		1.01 ± 0.14		0.94 ± 0.09		0.91 ± 0.07		0.90 ± 0.08		1.04 ± 0.07		0.96 ± 0.09	1.03 ± 0.12
		MV (mL)		143.20 ± 60.35		141.00 ± 37.66		123.00 ± 20.31		116.80 ± 24.18		113.80 ± 24.20		121.80 ± 22.40		127.20 ± 15.45		121.00 ± 14.75	146.80 ± 38.44
		RR (bpm)		170.40 ± 63.74		127.80 ± 24.24		124.00 ± 13.40		128.60 ± 26.02		130.20 ± 26.76		143.80 ± 32.28		127.80 ± 13.10		134.60 ± 33.19	147.00 ± 32.75

TV, tidal volume; MV, minute volume; RR, respiratory rate. Data were expressed as mean±SD, N=5. * and ** indicate significantly changed after administration (* *P* ≤0.05, ** *P* ≤0.01)

In summary, within the range of tested doses, the four-week repeated toxicity and respiratory toxicity studies demonstrated that the pulmonary administration of haMSC-EVs was safe.

### Therapeutic effects of haMSC-EVs in an LPS-induced rat model of ALI/ARDS via intratracheal atomization

**Fig. 6:**
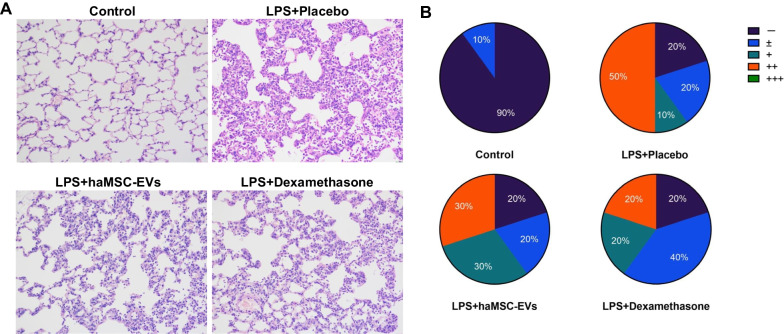
haMSC-EVs alleviated lung injury in an LPS-induced ALI/ARDS rat model. **A** Histopathology showed that haMSC-EVs decreased the infiltration of inflammatory cells in the alveolar lumen and interstitium and reduced the thickening of the alveolar septum. **B** haMSC-EVs alleviated the degree of lung lesions (n = 10). − indicates no lesions, ± indicates few lesions, + indicates mild lesions, and + + indicates moderate lesions, +++ indicates severe lesions

To verify the results of the in vitro potency assay, we tested the therapeutic effects of haMSC-EVs in an LPS-induced rat model of ALI/ARDS. The animals were randomly divided into four groups: the Control group, Placebo group, haMSC-EVs (6 × 10^7^ particles/rat/time for a total of 2 times) group, and Dexamethasone (1.6 mg/kg/time for a total of 2 times) group. After 24 h, haMSC-EVs reduced the incidence of moderate lung lesions in the model rats compared to that in the Control group (50% vs. 30%) (Fig. 6).

**Fig. 7:**
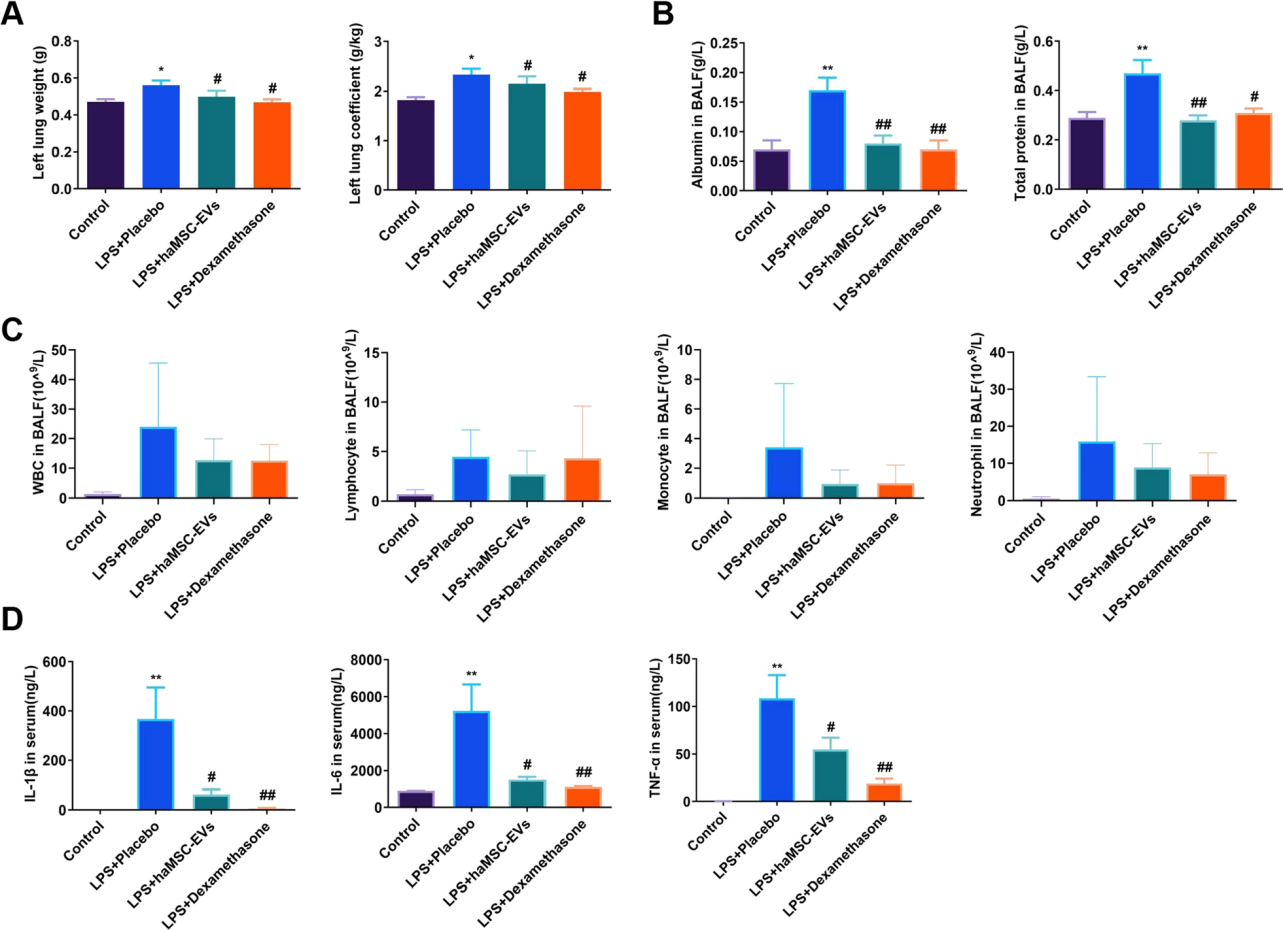
haMSC-EVs alleviated pulmonary edema and inflammation in an LPS-induced ALI/ARDS rat model. **A** At 24 h, the haMSC-EVs reduced the left lung wet weight and left lung coefficient. **B** haMSC-EVs reduced the serum ALB and total protein levels in BALF at 24 h. **C** Inflammatory cell count in BALF at 24 h. **D** Serum levels of IL-1β, IL-6 and TNF-α decreased 4 h after administration. The serum levels of IL-1β, IL-6 and TNF-α were below the detection limits 24 h after administration. * and ** indicate significant differences compared with the Control group (*, *P* ≤ 0.05; **, *P* ≤ 0.01). # and ## indicate significant differences compared with the LPS + Placebo group (#, *P* ≤ 0.05; ##, *P* ≤ 0.01)

To further understand the underlying mechanism, lungs tissues, BALF, and blood were collected for further analysis. At 24 h, compared with those in the Control group, the left lung weight and left lung coefficient in the Placebo group were significantly increased, suggesting pulmonary edema. haMSC-EVs significantly reduced LPS-induced pulmonary edema (0.561 ± 0.026 vs. 0.498 ± 0.033, *P* ≤ 0.05; 2.329 ± 0.119 vs. 2.144 ± 0.149, *P* ≤ 0.05) (Fig. 7A). haMSC-EVs also reduced the total protein and total ALB levels in BALF (0.5 ± 0.1 vs. 0.3 ± 0.0, *P* ≤ 0.01; 0.2 ± 0.0 vs. 0.1 ± 0.0, *P* ≤ 0.01) (Fig. 7B). The number of inflammatory cells in BALF was also significantly lower in the haMSC-EVs group than in the Control group (Fig. 7C). In addition, haMSC-EVs significantly decreased the serum levels of IL-1β, IL-6 and TNF-α at 4 h (367.5 ± 128.0 vs. 62.1 ± 20.6, *P* ≤ 0.05; 5222.0 ± 1441.6 vs. 1507.5 ± 153.1, *P* ≤ 0.05; and 108.4 ± 24.5 vs. 46.0 ± 9.8, *P* ≤ 0.05) (Fig. 7D). At 24 h, the serum inflammatory factor levels were below the detection limit.

Based on these results, we concluded that haMSC-EVs alleviated inflammation in LPS-treated rats mainly (or at least in part) by reducing the inflammatory factor levels in BALF and serum.

## Discussion

The therapeutic potential of EVs from different cell types for pulmonary infections has been reported. Zhu et al. reported that in the case of *E. coli* endotoxin-induced lung injury, endotracheal infusion of microvesicles derived from MSCs reduced the infiltration of alveolar proteins, reduced the lung tissue water content, and alleviated pulmonary edema. Moreover, the number of neutrophils and the level of macrophage inflammatory protein-2 in BALF were decreased. The team further reported that the transfer of microvesicle-loaded KGF mRNA to the alveolar epithelial cells played an important role in these therapeutic effects [24]. Recently, exosomes isolated from embryotic stem cell (ESC)- derived MSCs were shown to suppress complement-mediated neutrophil activation and inhibit the release of NETs and IL-17 by neutrophils. These processes inhibit the amplification and shorten the duration of inflammation during pulmonary infection [53].

In previous works, we described haMSC-EV products in detail from the aspects of the production process, quality control and so on [18]. In this study, we focused on the quantitative analysis and stability of the products, which are equally important to product quality. We established a relative quantification method with the nanoflow cytometry technique as suggested by the MISEV2018 and the existing literature [54, 55]. PKH67 was used to label the EV membranes in the products, and the relative quantitative analysis of EVs expressing different markers was carried out using specific antibodies. The results from multiple lots showed that PKH67-positive particles accounted for approximately 60% of the total number of particles, indicating that most of the particles in our product have a membrane structure. EVs secreted by different cell types express surface markers at different ratios [56]. According to our results, the percentage of haMSC-EVs positive for each surface marker was approximately 10%, and the consistency among lots suggested that our manufacturing process was stable. Considering the fluorescence resolution and labeling efficiency, the actual proportion of these marker-positive EVs may be higher.

While many studies on the storage conditions of EVs have been conducted, the results are mostly inconsistent, possibly due to the different cell sources and storage conditions of EVs. To address this issue, we designed stability tests based on the International Conference on Harmonization (ICH) guidelines (Q1A) and mimicked DP production scenarios. Changes in EV concentrations, marker expression and potency were evaluated. The results showed that the haMSC-EVs were stable for 6 months at − 80 °C and for 3 months at − 20 °C. In stress tests, we found that the properties and potency of the product did not significantly change within 6 h at room temperature. Because the low concentration in DP hinders the EV characterization, we are developing assays to characterize EVs in post-nebulized DS. And to assess the distribution of haMSC-EVs following nebulization, we conducted an aerodynamic study of EV substances using a next-generation impactor (NGI) (data not shown). Although the characterization of EVs still proved challenging due to the low concentration in the generated samples, we calculated the mass of EVs distributed in total and in droplets with diameters not exceeding 5 μm, representing fine particles capable of reaching and depositing in the lower lungs. The fine particle fraction (FPF) was determined to be 55.3%, and the median mass aerodynamic diameter (MMAD) was measured at 3.5 μm. These indicate that a significant portion of the particles could reach the lower respiratory tract. Although transcriptome and proteome changes are also important evaluation indices, the sampling method and repeatability of these methods limit their use as robust stability evaluation assays. The targeted detection of specific RNAs and proteins that have been shown to change in stability samples via electrophoresis may be more feasible. Moreover, a low temperature is still required for long-term storage, and formulation research is urgently needed.

MicroRNAs are important players in EV functions because they can achieve long-term intervention or treatment by regulating the expression of target proteins involved in disease-related signaling pathways. Studies have shown that microRNAs can regulate inflammatory signaling pathways by targeting the expression of specific proteins and mediating immune responses during lung injury [57]. Changes in microRNA profiles can affect the quality of haMSC-EVs. To verify the consistency of our product quality, we performed small RNA sequencing on 3 lots of haMSC-EVs. After normalization, the expression levels of highly abundant microRNAs were found to be consistent among the lots, and we discovered several microRNAs that have been reported to exert anti-inflammatory effects, such as miR-146a, miR-21-5p and let-7b. miR-146a, when stimulated by IL-1β, is packaged into EVs and transferred to macrophages, promoting the polarization towards the anti-inflammatory M2 phenotype. This leads to the suppression of systemic inflammation and an increase in the survival rate of septic mice [27]. In addition, hsa-miR-21-5p and hsa-let-7b were shown to suppress TLR4/NF-κB signaling by downregulating TLR4 expression, thus inhibiting the immune response to *Helicobacter pylori* infection or LPS-induced ALI [58, 59]. Quantification assays will be developed to evaluate specific contents of these microRNAs, and the therapeutic contributions of these microRNAs will be studied by conducting knockdown or knockout experiments. A proteomic study helped us verify the characteristics of the haMSC-EV products by comparing the data to the existing EV database. As expected, we identified 81 EV markers in our product samples and several MSC markers (CD90 and CD73; data not shown). Ninety percent of the proteins were consistently identified in the 3 lots, which suggested that the production process yielded EVs with consistent protein contents. We also found significant enrichment of inflammation-related pathways, such as those involved in complement and coagulation cascades, bacterial infection, and chemokine signaling, supporting the anti-inflammatory functions of haMSC-EVs.

Preclinical safety studies have shown that EVs from different cell types have suitable safety. A single-dose acute toxicity study using Expi293F cell-derived exosomes in mice showed no significant abnormalities in gross anatomy or histopathology, except for a slight change in blood cell count and a two-fold increase in neutrophils after 24 h. This suggests mild toxicity [60]. In repeated drug toxicity studies with fibroblast-derived exosomes in C57BL/6 mice over 4 months, mild inflammation was observed in various organs for both the treatment and control groups [39]. Similarly, a 3-week study with HEK293T-derived exosomes in C57BL/6 mice revealed minor pathological changes in the livers, indicating no drug-related abnormalities [61]. Most published studies evaluate the safety of intravenously administered EVs [40, 60, 61]. Intravenously administered MSC-EVs were reported to be mainly distributed in the liver, spleen, and kidney [62]. In comparison, in our previous study, we found that haMSC-EVs delivered through the trachea mainly accumulated in the lungs. Due to the differences in organ distributions caused by administration methods, we sought to evaluate the safety of pulmonary administered haMSC-EVs. We tested 3 doses starting with a 5-fold to 125-fold equivalent dose of the clinical initial dose (2 × 10^8^ particles/person) by intratracheal atomization [19]. Animal weight, gross anatomy, hematology indices, and immune function were compared between the control group and the treatment groups. Animal death and organ coefficient changes were occasionally observed but were not dose-dependent and were most likely caused by the operation or other disorders. Due to the intratracheal approach of administration, respiratory function indices were also carefully analyzed. The TV, MV, and RR were tested at different time points within 24 h after administration. The results revealed slight and occasional changes in TV (less than 1.5-fold) in both the control and treatment groups, which could be induced by intratracheal atomization. We also observed minor changes (less than twofold) in neutrophil and lymphocyte counts, which could be a result of normal physiological variation or interspecific administration, as reported in other EV safety studies [60]. To further verify this hypothesis, tests on animals from other species, especially primates, should be performed.

To investigate the in vivo therapeutic effects of pulmonary administered haMSC-EVs, EVs were intratracheally administered to rats with LPS-induced ALI/ARDS. We found that in the haMSC-EVs group, pulmonary edema, effusion, and cytokine levels in the serum were reduced, which suggested that the injury was alleviated. Although the lymphocyte changes were not significant, a decreasing trend was found for monocytes and neutrophils. Moreover, compared with those in the control group, the BALF IL-1β levels in the LPS + haMSC-EVs group were significantly lower at 24 h (*P* ≤ 0.01). Although the levels of IL-6 and TNF-α, showed a decreasing trend, they were not significantly different. One of the advantages of using a rat model is that blood gas analysis is applicable; therefore, we measured the arterial partial pressure of oxygen (pO_2_) and carbon dioxide (pCO_2_). Through this approach, we report that in the LPS group, pCO_2_ decreased after 24 h of administration, which was possibly caused by interstitial infiltration. This decrease led to a hypoxia-induced compensatory increase in the respiratory rate. In comparison, the haMSC-EVs group showed a rescue effects, which suggested improved lung function (Additional file 3: Table S3). The alleviation of inflammation and lung injury was consistent with published research [6, 43], suggesting that haMSC-EVs alleviate LPS-induced ALI/ARDS in rats mainly by reducing inflammation, which could be attributed to the related proteins and microRNAs within the EVs.

## Conclusions

In conclusion, the haMSC-EV production process reported in this study is stable, and haMSC-EVs are stable as off-shelf drugs. A QC system was established, and intratracheally administered haMSC-EVs demonstrated excellent safety at the tested dosages in systematic preclinical toxicity studies. Improved lung function and anti-inflammatory effects were observed in LPS-induced ALI/ARDS rats after the intratracheal administration of haMSC-EVs. While microRNA-seq and proteome analyses have provided insight into possible effective factors, verification and quantification assays should be further conducted.

### Supplementary Information

**Additional file 1**. ARRIVE Checklist.

**Additional file 2: Figure S1**. Original uncropped blots of the Western blot figure in the manuscript.

**Additional file 3: Table S1**. Effects of haMSC-EVs on cells in BALF of female rats. **Table S2**. Effects of haMSC-EVs on cells in BALF of male rats. **Table S3**. Arterial blood gas analysis in ALI model rats..

## Data Availability

The small RNA-seq data and proteomic data have been deposited in China National Center for Bioinformation / Beijing Institute of Genomics, Chinese Academy of Sciences under BioProject No. PRJCA016121 (https://ngdc.cncb.ac.cn/bioproject/browse/PRJCA016121) [63, 64]. All other data are included in the article and its Supplementary Information files or available from the corresponding authors upon reasonable request.

## Abbreviations

MSCs: Mesenchymal stromal cells
ALI: Acute lung injury
ARDS: Acute respiratory distress syndrome
EVs: Extracellular vesicles
haMSC-EVs: Human adipose mesenchymal stromal cells-derived extracellular vesicles
LPS: Lipopolysaccharide
BALF: Bronchoalveolar lavage fluid
ESC: Embryotic stem cells
QC: Quality control
CPPs: Critical process parameters
CQCPs: Critical quality control points
CQAs: Critical quality attributes
TMT: Tandem mass tag
LC–MS/MS: Liquid chromatography–tandem mass spectrometry
FBS: Fetal bovine serum
TEM: Transmission electron microscopy
NTA: Nanoparticle tracking analysis
NanoFCM: Nano-flow cytometry
GO: Gene ontology
KEGG: Kyoto encyclopedia of genes and genomes
ELISA: Enzyme-linked immunosorbent assay
TV: Tidal volume
MV: Minute volume
RR: Respiratory rate
GLP: Good laboratory practice
GMP: Good manufacturing practice
DS: Drug substance
DP: Drug product
RSD: Relative standard deviations
qPCR: Quantitative polymerase chain reaction
ChP: Chinese pharmacopoeia
BP: Biological processes
CC: Cellular components
MF: Molecular function
HGB: Hemoglobin
ALB: Albumin
