# Supplementary figures and images for: Manufacturing, quality control, and GLP-grade preclinical study of nebulized allogenic adipose mesenchymal stromal cells-derived extracellular vesicles
Source: Stem Cell Res Ther. 2024 Apr 2;15:95. doi: 10.1186/s13287-024-03708-1 (PMC10988864; doi:10.1186/s13287-024-03708-1)

**Supplementary figure**

**Figure S1**


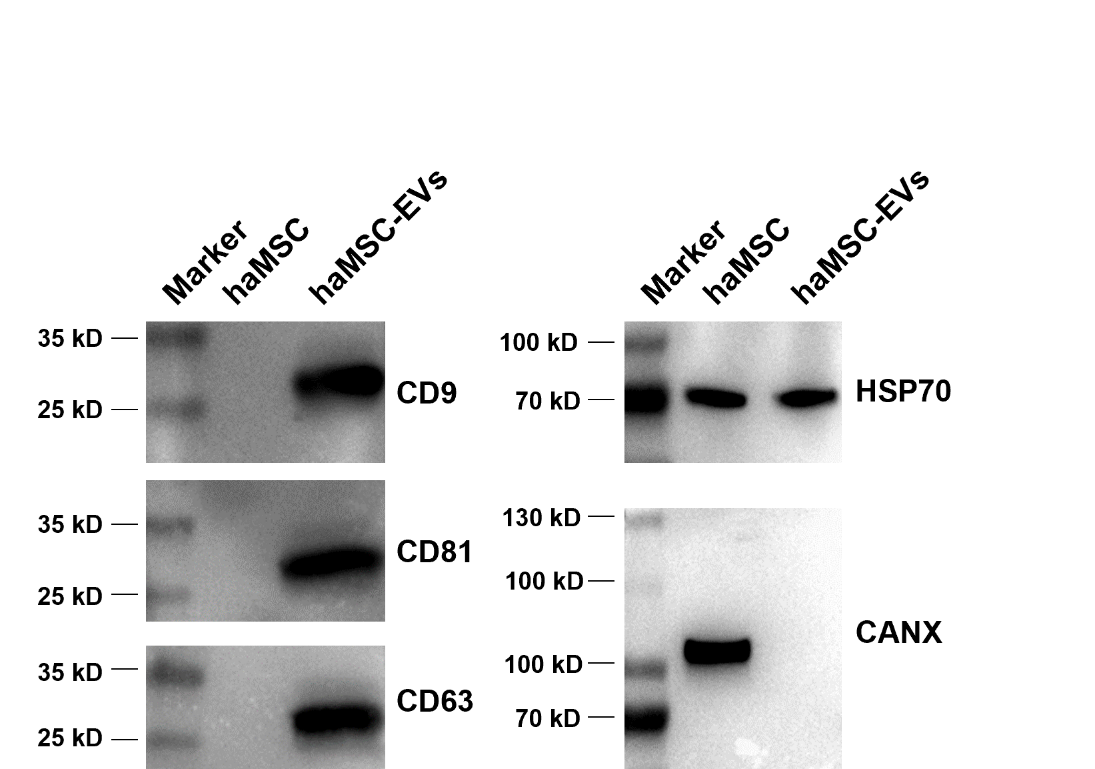


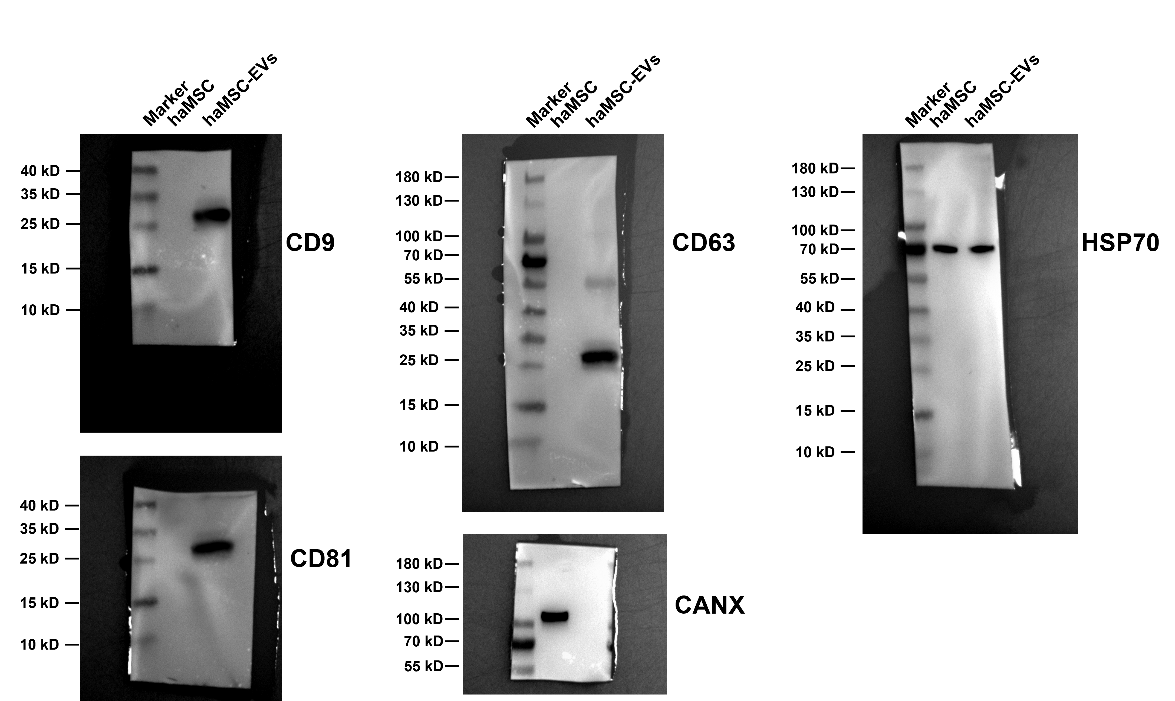

Supplement: Supplementary file 2 — Additional file 2: Figure S1. Original uncropped blots of the Western blot figure in the manuscript. [file 13287_2024_3708_MOESM2_ESM.docx]
