# Supplementary material for: Manufacturing, quality control, and GLP-grade preclinical study of nebulized allogenic adipose mesenchymal stromal cells-derived extracellular vesicles
Source: Stem Cell Res Ther. 2024 Apr 2;15:95. doi: 10.1186/s13287-024-03708-1 (PMC10988864; doi:10.1186/s13287-024-03708-1)
Supplement: Supplementary file 3 — Additional file 3: Table S1. Effects of haMSC-EVs on cells in BALF of female rats. Table S2. Effects of haMSC-EVs on cells in BALF of male rats. Table S3. Arterial blood gas analysis in ALI model rats.. [file 13287_2024_3708_MOESM3_ESM.docx]

| Time after administration | D29 | | | | D62 | | | |
| --- | --- | --- | --- | --- | --- | --- | --- | --- |
| Groups | Control | haMSC-EVs-low | haMSC-EVs-medium | haMSC-EVs-high | Control | haMSC-EVs-low | haMSC-EVs-medium | haMSC-EVs-high |
| Number of animals | 5 | 5 | 5 | 5 | 5 | 5 | 5 | 5 |
| #NEU (10^3 cells/µL) | 0.25±0.23 | 0.27±0.18 | 0.23±0.15 | 0.15±0.12 | 0.65±0.41 | 0.61±0.36 | 0.57±0.20 | 0.43±0.29 |
| #LYM (10^3 cells/µL) | 0.98±0.62 | 1.04±1.09 | 0.90±0.61 | 0.67±0.41 | 2.02±1.11 | 1.02±0.46 | 1.36±0.70 | 1.16±0.38 |
| #MON (10^3 cells/µL) | 0.01±0.01 | 0.02±0.02 | 0.02±0.02 | 0.00±0.01 | 0.04±0.02 | 0.04±0.02 | 0.05±0.02 | 0.04±0.03 |
| #EOS (10^3 cells/µL) | 0.01±0.01 | 0.01±0.01 | 0.01±0.00 | 0.00±0.01 | 0.02±0.02 | 0.02±0.01 | 0.01±0.01 | 0.01±0.00 |
| #BAS (10^3 cells/µL) | 0.05±0.04 | 0.06±0.05 | 0.03±0.02 | 0.02±0.01 | 0.12±0.09 | 0.07±0.02 | 0.07±0.03 | 0.06±0.02 |
| #LUC (10^3 cells/µL) | 0.16±0.14 | 0.19±0.14 | 0.16±0.10 | 0.10±0.10 | 0.33±0.18 | 0.31±0.23 | 0.31±0.09 | 0.24±0.21 |

**Supplementary tables:**

Table S1 Effects of haMSC-EVs on cells in BALF of female rats

#, absolute number of. NEU, neutrophil; LYM, lymphocyte; MON, monocyte; EOS, eosinophil; BAS, basophil; LUC, large unclassified cell. Data were expressed as‾X±SD, N=5. * and ** indicate significantly changed after administration (* P ≤0.05, ** P ≤0.01).

Table S2 Effects of haMSC-EVs on cells in BALF of male rats

| Time after administration | D29 | | | | D62 | | | |
| --- | --- | --- | --- | --- | --- | --- | --- | --- |
| Groups | Control | haMSC-EVs-low | haMSC-EVs-medium | haMSC-EVs-high | Control | haMSC-EVs-low | haMSC-EVs-medium | haMSC-EVs-high |
| Number of animals | 5 | 5 | 5 | 5 | 5 | 5 | 5 | 5 |
| #NEU (10^3 cells/µL) | 0.30±0.28 | 0.22±0.13 | 0.16±0.05 | 0.37±0.31 | 0.80±0.40 | 0.79±0.67 | 0.75±0.22 | 1.10±0.50 |
| #LYMY (10^3 cells/µL) | 2.17±1.62 | 0.92±0.74 | 1.38±0.43 | 2.24±1.62 | 2.84±1.78 | 2.23±1.28 | 2.98±2.79 | 1.87±0.85 |
| #MONO (10^3 cells/µL) | 0.02±0.02 | 0.01±0.02 | 0.02±0.02 | 0.02±0.02 | 0.06±0.02 | 0.07±0.04 | 0.05±0.02 | 0.07±0.03 |
| #EOS (10^3 cells/µL) | 0.02±0.01 | 0.01±0.01 | 0.01±0.01 | 0.02±0.02 | 0.03±0.02 | 0.02±0.01 | 0.02±0.01 | 0.02±0.02 |
| #BAS (10^3 cells/µL) | 0.04±0.01 | 0.03±0.02 | 0.03±0.01 | 0.05±0.03 | 0.09±0.03 | 0.09±0.05 | 0.12±0.14 | 0.10±0.01 |
| #LUC (10^3 cells/µL) | 0.09±0.04 | 0.09±0.06 | 0.07±0.02 | 0.13±0.08 | 0.44±0.24 | 0.35±0.30 | 0.46±0.23 | 0.68±0.50 |

#, absolute number of. NEU, neutrophil; LYM, lymphocyte; MON, monocyte; EOS, eosinophil; BAS, basophil; LUC, large unclassified cell. Data were expressed as‾X±SD, N=5. * and ** indicate significantly changed after administration (* P ≤0.05, ** P ≤0.01).

Table S3 Effect of haMSC-EVs on arterial Blood gas Analysis in ALI model rats

| **Time** | **Groups** | **pO2**  **（mmHg）** | **sO2**  **（%）** | **pCO2**  **（mmHg）** |
| --- | --- | --- | --- | --- |
| 4 h | Control | 60.4±3.2 | 86.3±2.5 | 58.6±3.2 |
|  | LPS+Placebo | 64.4±4.9 | 90.0±2.4 | 47.6±2.0** |
|  | LPS+haMSC-EVs | 68.0±3.0 | 91.9±1.2 | 43.8±1.1 |
|  | LPS+Dexamethasone | 59.6±3.1 | 87.6±1.9 | 48.7±1.6 |
| 24 h | Control | 61.7±4.0 | 90.0±1.2 | 48.5±1.1 |
|  | LPS+Placebo | 68.1±6.9 | 86.8±7.4 | 43.1±0.9* |
|  | LPS+haMSC-EVs | 68.8±3.2 | 92.2±0.9 | 48.1±1.0^△^ |
|  | LPS+Dexamethasone | 82.4±3.4^△^ | 94.6±0.7 | 55.9±1.2^△△^ |

* and ** indicate significantly changed compared with the control group (*, P≤0.05; **, P≤0.01). △ and △△ indicate significantly changed compared with the LPS+Placebo group (△, P≤0.05; △△, P≤0.01). pO2, arterial partial pressure of oxygen; sO2, oxygen saturation; pCO2, arterial partial pressure of carbon dioxide. Data were expressed as‾X±SD.
